# Milliwatt-level UV generation using sidewall poled lithium niobate

**DOI:** 10.1038/s41467-026-68524-y

**Published:** 2026-04-21

**Authors:** C. A. A. Franken, S. S. Ghosh, C. C. Rodrigues, J. Yang, C. J. Xin, S. Lu, D. Witt, G. Joe, G. S. Wiederhecker, K.-J. Boller, M. Lončar

**Affiliations:** 1 School of Engineering and Applied Sciences, Harvard University, Cambridge, MA USA; 2 Laser Physics and Nonlinear Optics, Department of Science and Technology, MESA+ Institute of Nanotechnology, University of Twente, Enschede, Overijssel the Netherlands; 3 Sabratha Photonics B.V., Enschede, Overijssel the Netherlands; 4 Department of Physics, Harvard University, Cambridge, MA USA; 5 Fermi National Accelerator Laboratory, Batavia, IL USA; 6 Gleb Wataghin Physics Institute, University of Campinas, Campinas, São Paulo Brazil

**Keywords:** Nonlinear optics, Nanophotonics and plasmonics, Nanophotonics and plasmonics

## Abstract

Integrated coherent sources of ultra-violet (UV) light are essential for a wide range of applications, from ion-based quantum computing and optical clocks to gas sensing and microscopy. Recently, approaches that use frequency upconversion have received considerable attention. Among these, the integrated thin-film lithium niobate (TFLN) photonic platform shows particular promise. However, to date, the high propagation losses and lack of reliable techniques for consistent poling of cm-long waveguides with small poling periods have impeded progress. Here, we present a sidewall poled lithium niobate (SPLN) waveguide approach that overcomes these obstacles and results in a two-orders-of-magnitude increase in generated UV power. We demonstrate SPLN waveguides featuring record-low propagation losses of 2.3 dB/cm, complete domain inversion across the waveguide cross-section, and an optimum 50% duty cycle, resulting in a record-high normalized conversion efficiency of 5050%W^−1^cm^−2^, and 4.2 mW of generated on-chip power at 390 nm wavelength. This advancement makes the TFLN platform a viable option for high-quality on-chip UV generation, benefiting emerging applications.

## Abstract (web summary)

Integrated ultra-violet (UV) light sources are essential for applications from quantum computing to sensing. Here, authors demonstrate a two-orders-of-magnitude increase in UV power generation through efficient frequency up-conversion in record-low loss, sidewall poled lithium niobate waveguides.

## Introduction

A wide range of emerging technologies, including ion trap-based quantum computers^1^, optical clocks^2^, super-resolved structured illumination microscopy^3^, and spectroscopy^4^ rely on efficient generation and delivery of coherent optical signals at ultra-violet (UV) wavelengths. Unfortunately, semiconductor laser diodes in this wavelength range are either not readily available or do not meet the tunability and linewidth requirements^5–7^. To meet the demands of such applications, novel hybrid and heterogeneously integrated laser sources have been developed in the visible violet range^8,9^. However, straightforward extension into the UV currently remains difficult due to the lack of suitable semiconductor amplifiers.

Alternatively, nonlinear conversion based on bulk crystals like *β*-barium borate^10^ and lithium tantalate^11^ has been leveraged to realize robust, wavelength agile UV sources. Lithium niobate (LN) is another promising candidate, with a high optical nonlinearity and comparably large transparency window down to 350 nm^12^, which has facilitated efficient frequency conversion using bulk LN periodically poled waveguides^13,14^. UV generation has been demonstrated in bulk and thin-film LN using resonantly-enhanced second harmonic generation (SHG) in nanospheres^15^, Cherenkov phase matching at an LN-BBO interface^16^, and supercontinuum generation^17,18^, albeit at low powers.

Recently, the ultra-low loss thin-film lithium niobate (TFLN) photonic platform has emerged as a powerful approach to realize high performance electro-optic (EO) devices and chip-scale systems at infra-red and visible wavelengths^19,20^. In addition, frequency comb sources spanning UV-to-visible ranges have been demonstrated in TFLN^21^. State-of-the-art periodically poled TFLN waveguides have been used to produce up to 30 μW of on-chip UV power through second harmonic generation^22^. However, this output is significantly lower than what is theoretically achievable, given the material’s high nonlinearity and the enhanced interaction enabled by the tight confinement of TFLN waveguides. The lower than expected efficiency of this implementation has been attributed to high propagation losses (7.6 dB/cm), strong deviation from the optimum duty cycle (90% instead of ideal 50%), and film thickness variations throughout the waveguide. Additionally, the rate of domain inversion via this poling method is highly sensitive to variations in the poling field^23,24^. Addressing all these issues while achieving the small μm-order poling period throughout a cm-scale waveguide is among the greatest challenges for generating UV in TFLN at mW-level powers. Only in that power regime do such sources become practically relevant for applications such as quantum computing^5^.

Besides lowering propagation losses, recent progress on the standard pole-before-etch method, as used by Hwang et al.^22^, can improve UV conversion efficiencies. Chen et al. report that adapting the poling period to account for film thickness variation greatly increases the efficiency for infra-red to visible upconversion^25^. However, this method still does not account for the fabricated geometry, i.e., waveguide shape and etch depth variations. More recent work addresses this by poling the film after etching the waveguide and taking such variations into account^26^. The drawback of this pole-after-etch approach is the placement of the poling fingers on the etched part of the film (slab), only inverting the slab part of the waveguide cross-section. Since a large fraction of the optical mode resides in the unetched part of the waveguide (ridge), this partial inversion can reduce the efficiency to as low as 20% of the maximum achievable efficiency in a completely inverted film^26^. The partial inversion can be leveraged to facilitate inter-modal phase matching, but remains below adapted poling efficiencies^27^. Adjusting the placement of the poling fingers in pole-after-etch to achieve complete inversion has not been explored. However, earlier work at infra-red wavelengths has placed electrodes closer to the optical mode to improve local control over poling in bulk-LN^28^, or to avoid the propagation loss that can occur in pole-before-etch waveguides due to differential etching of poled domains^29^, but efficiencies have remained low, not exceeding 500%W^−1^ cm^−2^.

**Fig. 1:**
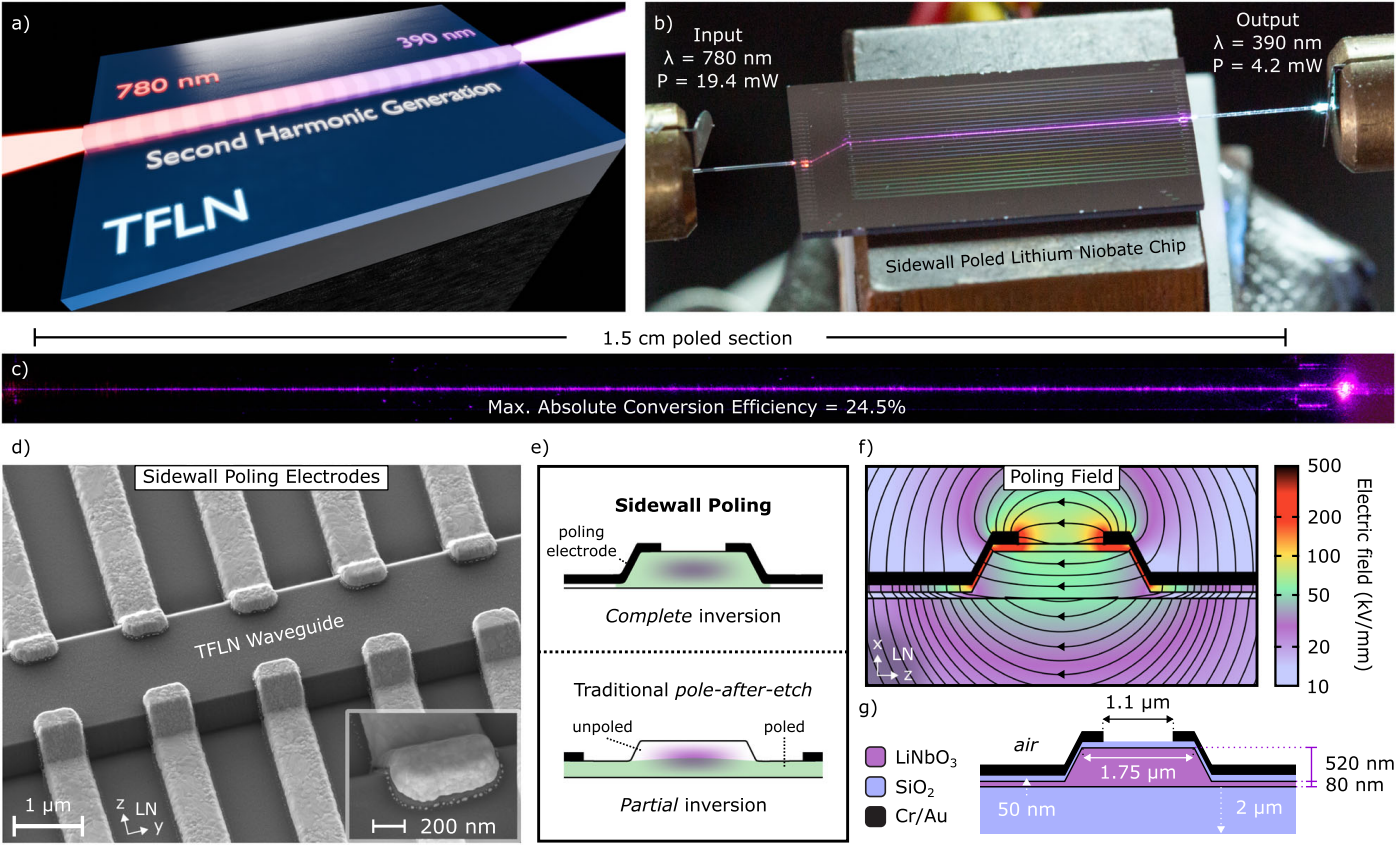
UV generation using sidewall poled lithium niobate (SPLN) waveguides. **a** A second harmonic generation (SHG) process is used to upconvert visible 780 nm light to the ultra-violet (UV) at 390 nm. The quasi-phase matched nonlinear process is enabled by periodically inverting the sign of the *χ*^(2)^ nonlinearity in the thin-film lithium niobate (TFLN) waveguide using our sidewall poling method. **b** Image of the sidewall poled lithium niobate chip recorded during UV generation. Specified powers indicate on-chip values. **c** Top-down microscope image of the 1.5-cm long SPLN waveguide; here, the UV light is being generated as the pump propagates through the waveguide from left to right. **d** Scanning electron microscope image of metal electrodes used for sidewall poling. The electrodes reach from the TFLN slab (etched portion of the film) onto the waveguide ridge. By placing the poling fingers onto the waveguide, the duty cycle can be precisely controlled over long waveguide sections while ensuring that the entire waveguide cross-section is poled, thus realizing efficient conversion to the UV. Once established, the domain inversion remains stable, allowing for the removal of the metal electrodes to minimize optical losses. **e** Schematic showing a benefit of sidewall poling vs. traditional pole-after-etch methods; namely, complete inversion of the waveguide, a feature that can increase the normalized conversion efficiency significantly. **f** Simulation of the electric field pattern resulting from 127-V potential difference applied to the poling electrodes. Note that the field lines are nearly parallel to the crystalline *z*-axis, and, more importantly, the field strength in the entire waveguide cross-section is beyond the coercive field strength needed for domain inversion in thin-film lithium niobate (~30 kV/mm)^53^. **g** Materials and dimensions for the waveguides and sidewall poling electrodes discussed in this work.

Here we present an improvement by two orders of magnitude over the state-of-the-art in UV power^22^, reaching the mW-level for the first time, using sidewall poled lithium niobate (SPLN) waveguides and second harmonic generation (Fig. 1). Key to our pole-after-etch approach is the precise placement of the poling electrodes onto the sidewalls of the MgO-doped TFLN waveguide in combination with adapted poling^25,26^. This allows us to take fabrication-induced variations to the waveguide geometry into account while ensuring that each poled region is fully inverted across the entire waveguide cross-section, thus overcoming a major drawback of the pole-after-etch approach^26,30^. Using comb-to-comb shaped poling electrodes with flat fingers positioned on the waveguide sidewalls to a precision of 50 nm, we achieve close to optimal (i.e., 50%) duty cycle along 1.5-cm-long waveguides and a reproducible robustness to poling field variations. These factors, combined with low UV loss (2.3 dB/cm), high confinement waveguides result in a record-high nonlinear conversion efficiency of 5050%W^−1^ cm^−2^ with a maximum 4.2 mW of generated on-chip UV power.

## Design and poling process

**Fig. 2:**
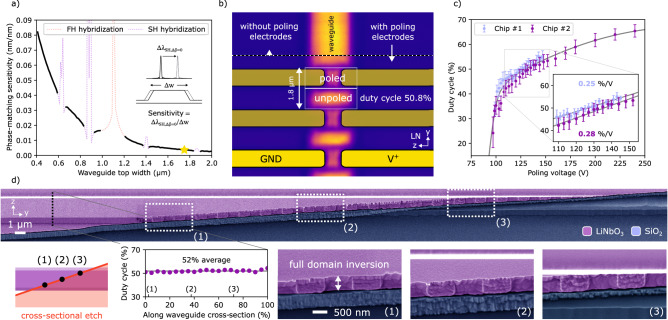
Optimization of the poling process. **a** Phase-matching sensitivity, defined as the derivative of the optimally phase matched second harmonic wavelength with respect to waveguide top width, for different waveguide top widths. In other words, the sensitivity captures the change in the phase matched SH wavelength (at *Δβ* = 0) as a function of variation in waveguide width. It can be seen that the phase-matching condition for a wide waveguide is more resilient to fabrication tolerances that result in width variation. Our waveguide width of 1.75 μm (indicated by a star) minimizes phase-matching sensitivity to near zero, while avoiding divergences in the sensitivity originating from transverse mode hybridization (i.e., hybrid modes with a TE polarization below 90%). **b** Second harmonic microscope image showing sidewall poled domains in the waveguide ridge. The boundaries between inverted domains appear as dark horizontal stripes in the image. The poling electrodes are indicated in yellow. For the inverted domain in this image, a near-optimum duty cycle is verified at 50.8%. **c** Dependence of duty cycle on applied poling voltage for two fabricated chips. Each data point corresponds to a single test structure that is poled at a different poling voltage. At lower voltages below 100 V the duty cycle falls off to the threshold voltage, and at higher voltages, the duty cycle saturates. The solid line based on a spline interpolation is provided as a visual reference, and the error bars indicate the experimental error. **d** Colored scanning electron microscope images show that a diagonal cross-sectional cut through the waveguide that has been exposed to a differential wet etch reveals the domain inversion extending completely through the waveguide cross-section, from top to bottom. The duty cycle along the diagonally etched waveguide cross-section is inferred from the SEM image and plotted. A constant duty cycle indicates parallel domain walls, ensuring a constant phase-matching condition across the entire mode.

Achieving a device design with high conversion efficiency starts by determining the optimal waveguide geometry (Fig. 1g). Recent work has highlighted that film thickness variations can negatively impact the phase-matching conditions and efficiency^26,31^. Therefore, it is important to consider waveguide geometries that minimize the sensitivity of the phase-matching condition to film thickness variation, such as waveguides etched in thicker films^22,32^. Besides lower sensitivity, thicker waveguides provide higher confinement and lower sidewall scattering loss, which can ultimately result in a higher conversion efficiency. Both considerations motivate our choice of a 600-nm thick lithium niobate film. Our choice of etch depth, around 500 nm, ensures full penetration of the poling field into the waveguide when using sidewall electrodes. In our work, any remaining sensitivity to film thickness and etch depth variations is mitigated by using adapted poling^26,31^. As a result, waveguide width variation becomes the dominant factor impacting phase matching^32^. Therefore, we investigate phase-matching sensitivity to waveguide width variations in order to determine an optimal waveguide geometry. In Fig. 2a we plot phase-matching sensitivity, here defined as the derivative of the optimally phase matched second harmonic wavelength with respect to waveguide top width, for a range of waveguide widths (see “Methods”). The resulting curve shows that the phase matching in wider waveguides is more robust to width variation from fabrication error. It can be seen that the chosen waveguide width of 1.75 μm minimizes phase-matching sensitivity to near zero, while avoiding divergences in the sensitivity originating from transverse mode hybridization.

Compared to traditional pole-after-etch methods, sidewall poling positions electrodes such that they extend from the slab up onto the waveguide sidewall (Fig. 1d, e), yielding several benefits. Simulation of poling electrodes on the waveguide sidewalls shows a uniform electric poling field across the entire waveguide which indicates homogeneous poling throughout the waveguide cross section (Fig. 1f, calculated using a finite element mode solver), thereby realizing the complete cross section domain inversion which maximizes conversion efficiency (Fig. 1e). The 1.75 μm waveguide width also tightly confines the fundamental and second harmonic modes in the waveguide ridge, reducing sidewall scattering significantly (see Supplementary Information) while maintaining a high overlap between the interacting modes (98%) for efficient conversion. While this waveguide width allows propagation of higher-order transverse modes at 390 nm, only the fundamental transverse mode at 780 nm is injected, and the poling is designed to only phase match the fundamental TE-polarized modes at 780 and 390 nm. The fabrication process of the waveguide and poling electrodes is described in “Methods”.

To ensure an optimum poling duty cycle (50%), poling is undertaken iteratively, alternating between applying electrical pulses to poling electrodes and imaging the poled structures using a high-resolution second harmonic microscope (details in “Methods”). Such images show dark lines between the inverted domains (Fig. 2b), which allows for a straightforward extraction of the poling duty cycle (see Supplementary Information). The duty cycle as a function of poling voltage is characterized on two separately fabricated chips with the same design (Fig. 2c). This measurement identifies an optimal poling voltage around 127 V, and shows that the duty cycle is robust to voltage variations: the duty cycle remains within 40–60% when the poling voltage is within ±20% of the optimal voltage (Fig. 2c inset). The poling duty cycle as a function of voltage also shows nearly the same slope in the 50% range across both chips, highlighting the reproducibility with which optimal duty cycle can be achieved with sidewall poling electrodes.

In order to verify the quality of poled domains, a diagonal cross-section of an SPLN waveguide is exposed by etching a trench through the waveguide after poling. The domains are revealed by immersing the waveguide in an etchant which etches LN crystalline z-faces at different rates (details in “Methods”)^26^. The trench is imaged using scanning electron microscopy (SEM) (Fig. 2d). The images show that the inverted domains extend uniformly across the waveguide ridge as well as all the way into the slab below the ridge. This contrasts with earlier poling techniques that position electrodes only on the slab, restricting domain inversion to the slab region (Fig. 1e). The experimentally inferred duty cycle throughout the waveguide cross-section reveals little variation (Fig. 2d plot). This indicates straight and parallel domain walls, ensuring a constant phase-matching condition across the entire mode.

## Results

**Fig. 3:**
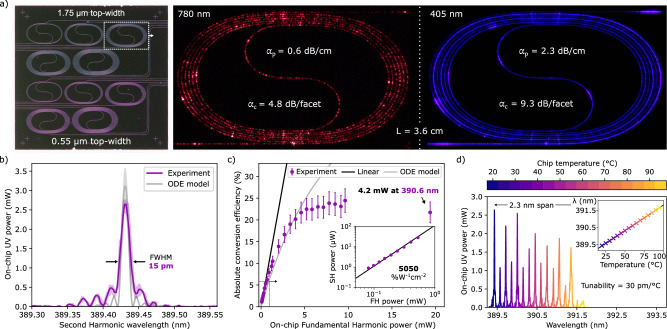
Characterization of linear and nonlinear properties of fabricated waveguides. **a** A set of spiral waveguides is used to characterize the propagation (*α*_*p*_) and fiber-to-chip coupling (*α*_*c*_) losses near the fundamental and second harmonic wavelengths, at 780 nm and 405 nm respectively. The record-low propagation loss at 405 nm is attributed to the wide waveguide used, which reduces sidewall scattering loss. **b** The phase-matching function measured by sweeping the fundamental harmonic wavelength and recording the second harmonic signal. The narrow peak indicates a constant phase-matching condition along the length of the 1.5-cm long waveguide and is consistent with the width predicted by our ordinary differential equation (ODE) model. Deviations from the expected sinc^2^-shape function (ODE model) are attributed to residual fabrication errors in the waveguide shape along the waveguide length. The shaded area surrounding the solid lines denotes the deviation in UV signal for both the experiment and theoretical model data. **c** Absolute UV conversion efficiency as a function of on-chip power of the fundamental harmonic. At approximately 10 mW of input, the conversion efficiency peaks at 24.5%, and a record-high on-chip UV power of 4.2 mW is recorded at maximum input power. The grey curve (ODE model) represents a theoretical model that accounts for pump depletion and includes both linear and nonlinear losses (details in main text and “Methods”). The inset double-logarithmic plot shows the power in the undepleted regime, with a quadratic fit (linear in log-space) indicating a high undepleted conversion efficiency of 5050%W^−1^ cm^−2^. The purple error bars indicate the experimental error, and are equal to the size of the markers for the inset plot shown at logarithmic scale. **d** The phase-matching function as a function of a chip temperature. A 2.3 nm-wide wavelength (*λ*) tuning range can be achieved.

To accurately estimate the on-chip fundamental and second harmonic powers, we experimentally evaluate the waveguide coupling and propagation losses at 780 and 405 nm, respectively, with a set of spiral waveguides (Fig. 3a), fabricated using the same process as the SPLN waveguides. The measurements (details in Supplementary Information) show a fiber-to-chip coupling loss of 4.8 dB/facet and a propagation loss of 0.6 dB/cm at 780 nm, achieving a performance comparable to state-of-the-art implementations of visible TFLN waveguides in thinner films than the present work^33^. A fiber-to-chip coupling loss of 9.3 dB/facet and a record-low propagation loss for TFLN waveguides of 2.3 dB/cm is measured at 405 nm.

Next, we experimentally map the phase-matching function (Fig. 3b), thereby determining the wavelength at which second harmonic generation is maximized. The second harmonic signal as a function of input wavelength is measured by coupling 780 nm light from a mode-hop-free tunable CW laser into the waveguide. At the output of the SPLN chip, the transmitted light is filtered to exclude the fundamental harmonic before the second harmonic signal is detected (see “Methods”). The measured phase-matching function features a sinc^2^-like shape, as predicted by our theoretically derived model, with a narrow (15 pm linewidth) central peak. This indicates that the nonlinear interaction has a near-constant phase-matching condition along the entire length of the waveguide, and that the negative impact of fabrication variations is mitigated by adapted poling and our waveguide design. The model is based on a system of coupled mode equations for the second harmonic generation process, including terms that account for pump depletion and both linear and nonlinear losses, and is discussed with further context below. The slight discrepancy between the measured and theoretically predicted profiles in the sidelobes can be attributed to waveguide geometry imperfections that occur during fabrication and are unaccounted for in the adapted poling process. With the pump wavelength tuned to the peak of the phase-matching function, the increase of UV power along the propagation length is sufficient for scattered UV light throughout the waveguide to be detected by a camera (Fig. 1b, c).

In order to characterize the normalized conversion efficiency of the sidewall poled waveguides, the pump wavelength is tuned to the peak of the phase-matching function, and the second harmonic signal is measured as a function of input power. At sub-mW pump powers, a linear increase in absolute efficiency as a function of pump power is observed (Fig. 3c). In this undepleted pump regime, a record-high normalized efficiency of 5050%W^−1^ cm^−2^ is measured (3c inset). A theoretically achievable normalized conversion efficiency of 9048%W^−1^ cm^−2^ is calculated using the measured propagation loss at UV and visible wavelengths and nonlinear couplings determined from first principles (details in “Methods”). This is a factor of ~1.8 larger than the experimentally measured value, with the discrepancy likely due to a residual phase mismatch.

Increasing the fundamental harmonic power to around 10 mW, a maximum absolute efficiency of 24.5% is measured. Increasing the power even further, we demonstrate a peak on-chip UV power of 4.2 mW at 390.6 nm, an improvement by more than two orders of magnitude over the state-of-the-art^22^. We note that this is the first demonstration in TFLN of UV generation at optical powers that exceed the milliwatt level. Accordingly, the leveling of the absolute efficiency in the higher power regime, exceeding pump powers of 3 mW, has not been observed before in this platform. We study this by modeling the interacting field amplitudes using a pair of coupled, ordinary differential equations (ODEs) that describe the second harmonic generation process (see “Methods”). The model incorporates a fit to the normalized conversion efficiency, measured linear losses, and reported values for UV and visible two-photon absorption (TPA) coefficients in bulk lithium niobate^34,35^. Using these parameter values and their reported uncertainties, the model describes the data up to 5 mW of input power (Fig. 3c), which is well beyond the undepleted, linear regime. The discrepancy beyond 5 mW is attributed to the fact that the nonlinear loss mechanisms and related effects have not been experimentally characterized in MgO-doped TFLN. A free fit of all TPA coefficients is also explored and matches the experimental data up to higher powers (see Supplementary Information), but gives values which deviate significantly from those previously reported in literature. Therefore, an opportunity is highlighted here for further material investigation in this new wavelength and intensity regime.

## Discussion

Using advanced poling strategies for TFLN and an optimized waveguide geometry, we have demonstrated more than 4 mW of UV power generated on-chip, exceeding the state of the art by more than two orders of magnitude. This is enabled by a record-high visible-to-UV conversion efficiency of 5050%W^−1^ cm^−2^. Our novel sidewall poling approach allows for complete (100%) domain inversion across the TFLN waveguide cross-section using a pole-after-etch method that typically can only invert the waveguide slab^26^. This is particularly effective for waveguide geometries considered in this work that feature very thin slabs (80 nm) and a thick waveguide ridge (520 nm). An early study on different film stacks shows that a 300-nm etched ridge waveguide, on a 600-nm film, can also be completely inverted using sidewall poling albeit at higher poling voltages (details in Supplementary Information). Besides complete domain inversion, we also show that sidewall poling results in straight and parallel domain walls. Precise poling control enabled accurate targeting of the optimal (50%) poling duty cycle at low poling voltages, verified at multiple locations along the 1.5-cm length waveguide. A saturation of the poling duty cycle—that is, its insensitivity to the precise voltage applied—was observed at higher voltages, which can further simplify the poling process. For example, by pre-compensating the width of the poling fingers, a duty cycle around 50% could be achieved for a wide range of voltages exceeding the saturation voltage. This reduces time-consuming poling calibration steps that rely on the iterative process of poling and imaging of the poled devices.

The high conversion efficiency we demonstrated is also enabled by the record-low loss of 2.3 dB/cm measured near the UV. This propagation loss is comparable to or even lower than that measured in waveguides based on materials with much wider transparency windows, such as Al_2_O_3_ and AlN^8,36,37^. The low propagation loss is attributed to our choice of wide and thick waveguides. While narrower waveguides have an increased mode intensity and thus increased nonlinear interaction^38^, they suffer from increased phase-matching sensitivity (Fig. 2a) and increased losses from sidewall scattering (see Supplementary Information). For example, we measured the propagation loss of 0.55-μm wide waveguides at 405 nm using fabricated spirals (Fig. 3a) and found a loss value of 5.4 dB/cm. This is much larger than the 2.3 dB/cm measured in the case for the 1.75-μm wide waveguides used in this work. As a result, the narrower waveguides have lower theoretical conversion efficiency (8699%W^−1^ cm^−2^) than the wide ones (9048%W^−1^ cm^−2^). This finding was also validated experimentally (Supplementary Information). Further reduction of the sidewall roughness of the waveguides could help decrease the propagation loss and, consequently, increase the efficiency for narrow waveguides^39^.

Our poling approach takes into account the film thickness of the starting LN film and etch depth variations^25,26^. Using this strategy combined with sidewall poling, we obtain a phase-matching function close to the ideally expected sinc^2^-function, indicating high-quality poling. Notably, we achieve a 24.5% absolute conversion efficiency—the highest ever reported for UV generation in this platform—entering a new regime where nonlinear absorption becomes relevant for the first time. The new regime is indicated by a plateau of the conversion efficiency at higher pump powers (Fig. 3c). We find that our first model using literature values for nonlinear losses (e.g., TPA) matches our experimental data up to about 5 mW. A free fit of model parameters yields a better agreement between experiment and theory (see Supplementary Information), but may result in best-fit TPA coefficients that deviate from literature values. To understand the nonlinear loss mechanisms in MgO-doped TFLN at these wavelengths and intensities, a more in-depth material study is needed. Such studies can also shed light on mechanisms that can help reduce the efficiency-diminishing effect of nonlinear losses, such as pulsed pump operation^34,35^ or optimized waveguide geometries.

The mW-level UV generation demonstrated here is sufficient for a wide range of applications from integrated ion traps to microscopy and spectroscopy^1–4^. The wide transparency of TFLN, low device complexity, and high reproducibility make sidewall poled waveguides in TFLN an excellent candidate for integrated UV sources. While semiconductor laser diodes in the UV require complex process development for shifting the wavelength of interest^6,7^, SPLN waveguides can be adapted for a broad range of wavelengths by simply changing the poling period. Furthermore, by heating the SPLN chip, we demonstrate the ability to tune the phase-matching function across a 2.3 nm wavelength range, with a tuning efficiency of 30 pm/°C as expected from literature (Fig. 3d)^22^. The rapid variation in the phase-matching function height with respect to temperature is attributed to mode crossings at elevated temperatures, and the overall trend of decreasing height of the phase-matching function (and increasing width) is attributed to the change in material dispersion with temperature^40^. UV wavelengths beyond the capabilities of our device should be possible straightforwardly using our approach down to 350 nm, limited by the band gap of lithium niobate^12^, albeit with lower efficiency due to increased absorption losses and challenges associated with realizing small poling periods using a lift-off process. Applying sidewall poling to the emerging thin-film lithium tantalate platform, featuring an enhanced photorefraction and optical damage threshold but also a reduced nonlinear coefficient, might become the next step for UV conversion down to 315 nm^41,42^; indeed, conventional periodic poling and second harmonic generation of green light have been demonstrated in this platform^43,44^. While the required shorter UV poling periods are also in reach with standard pole-before-etch techniques (~800 nm)^24,45^, ultra-short poling periods (~200 nm) were most recently achieved, after appearance of our pre-print, using our sidewall poling method enabling efficient backward propagating nonlinear conversion^46^. Besides wavelength versatility, UV SPLN can potentially bring linewidth reduction to coherent on-chip UV sources where currently intracavity losses limit the laser linewidth^8,9^. By upconverting visible on-chip lasers, which have widely demonstrated ultra-narrow linewidths^47–50^, the laser linewidth is largely transferred to the UV, and highly coherent, fully integrated UV sources can be realized^51^.

## Methods

### Nanofabrication and poling

Alignment markers are first patterned in S1822 resist deposited on a 5% MgO-doped, x-cut TFLN chip using optical lithography, and then dry-etched into the lithium niobate film. Prior to waveguide patterning, an aligned ellipsometry measurement is performed on a dense grid of points along the intended locations of the waveguides to assess the film thickness non-uniformity. The waveguides and sets of local alignment markers are then patterned using aligned e-beam lithography on negative maN-2405 resist and transferred to the lithium niobate film with a reactive Ar-ion etch. A post-fabrication cleanup is performed in heated SC-1 (60 °C) to remove etch redeposition, and another aligned ellipsometry measurement is performed to assess the etch depth variations along the chip. To prevent metal contamination of the film and electrically isolate the poling electrodes^52,53^, a thin protective SiO_2_ interlayer is deposited using inductively coupled plasma chemical vapor deposition.

Using pre-etch and post-etch ellipsometry data, the adapted poling period required for type-0 second harmonic phase matching is calculated, and poling electrodes are designed. For the waveguide reported here, the poling period varies from 1.799 μm to 1.814 μm. To account for lateral spreading of the inverted domains during poling, the width of the poling electrodes is set to 35% of the poling period. The poling electrodes (Cr/Au) are then fabricated using a lift-off process (LOR 3A and ZEP520A). The electrodes are patterned with an aligned e-beam write using local alignment markers to ensure precise alignment of the entire pattern with the pre-fabricated waveguide ridges at a tolerance of ≤50 nm. After developing, the electrodes are defined using e-beam evaporation and lift-off. First, an adhesion layer of chromium (25 nm) is deposited, followed by a layer of gold (125 nm). Following this step, the remaining resist is lifted off to define the electrodes. A layer of photoresist (S1822) is deposited on top of the electrodes to prevent breakdown during the poling, and contact windows for the poling probes are defined using photolithography. Poling is performed by touching probes onto the exposed pads and by applying a voltage pulse. The poling pulse parameters that are used can be found in ref. ^26^. The pulses are generated by a digital-to-analog converter and then amplified to reach the required voltage. Non-destructive imaging using oil-immersed second harmonic microscopy is used to verify the poling at a high resolution, for at least 3 locations along each 1.5-cm long waveguide.

After poling, the chip facets are defined using a deep etching process. First, the protective photoresist is removed by immersing the sample in Remover PG, the electrodes are etched away in gold etchant, followed by chromium etchant, and finally, the oxide interlayer is removed using diluted hydrofluoric acid. Then, 8.5-μm thick photoresist (SPR-220-7.0) is deposited, and boundaries for the deep etch are defined using photolithography. After developing, the lithium niobate and buried oxide are fully etched through at the facet locations using a reactive ion etch, and the silicon handle is etched through using a Bosch process etch^54^. Finally, any remaining resist is removed from the singulated chip by immersing it in Remover PG. The chip is finally annealed for 2 hours at 520°C in an oxygen atmosphere to reduce absorption losses.

To inspect the quality of our poling, we produce the structure as in Fig. 2d using the following process. First, a thin SiO_2_ layer is deposited on the SPLN waveguide using plasma enhanced chemical vapor deposition. Next, a diagonal pattern is defined using photolithography at an angle that should cross the waveguide width in about 20 poling periods and etched using reactive ion etching. To expose the inverted and non-inverted domains in the SPLN waveguide, revealed by the diagonal etch, the sample is immersed in heated SC-1 at 65 °C, taking advantage of the fact that SC-1 etches the faces of inverted and non-inverted regions of the crystal with different rates. Next, the protective SiO_2_ layer is removed in diluted hydrofluoric acid, and the sample is imaged using scanning electron microscopy.

### Experimental set-up for UV SPLN characterization

The SPLN chip is mounted on a copper stage that is temperature controlled using a Peltier element and a thermistor. During the measurement of the phase-matching function (Fig. 3b), the chip was at a temperature of 20 °C. For all data points in Fig. 3c the chip was set to 65 °C. Fiber-coupling for the input and output facet is achieved with lensed PM-630HP fibers (OZ Optics) and two high precision, three-axis stages (Thorlabs NanoMax 300). We do not find any difference measuring power or phase-matching function curves using this fiber (effectively multimode for UV) and a single mode UV fiber (Thorlabs SM300). This indicates that our UV generated light is dominated by the single TE_00_ mode. We chose to perform measurements with PM-630HP instead of a single mode UV fiber because the former also guides the pump wavelength, and thus can facilitate setup alignment even in the absence of UV light. No photodarkening or solarization of this fiber was observed during the experiments. The pump power exiting the lensed fiber and incident on the chip is measured with a calibrated visible range photodiode (Thorlabs S120C, with fiber mount) and the UV power at the output fiber is collected using a calibrated UV photodiode (Thorlabs S120VC, with fiber mount); for the latter the pump is blocked with a filter (OD  > 7 at 780 nm, 405/150 nm BrightLine single-band bandpass filter). For measurements of the phase-matching function a variable gain amplified photodiode (Thorlabs PDA100A2, with fiber mount) is used in combination with the previously mentioned pump filter to measure the UV power. The phase-matching function is recorded by sweeping the pump laser (CW, Toptica DL Pro 780 nm), while recording the laser wavelength and power (HighFinesse Wavelength Meter WS-7), and the UV signal on the photodiode using a digital-to-analog converter (National Instruments USB-6210). The pump laser can be swept mode-hop-free reliably over about 85 GHz frequency range, using both piezo and current control of the laser. The recording for a typical phase-matching function measurement spans about 400 GHz and is collected by stitching mode-hop-free sweeps together while rotating the grating of the laser simultaneously (with an automated motor control). The data analysis method to obtain the phase-matching function from the raw measurement data can be found in the Supplementary Information. For the measurements of the absolute conversion efficiency as a function of pump power (Fig. 3c), a variable optical attenuator is used (Thorlabs V600PA), except for the highest pump power measurement. The insertion loss of the attenuator is about 3 dB, explaining the gap between the highest and second-highest fundamental harmonic power data points in Fig. 3c.

### Phase-matching sensitivity calculation

For a given periodic poling grating momentum *G* and waveguide top width *w* the phase matched second harmonic generation wavelength is whichever second harmonic wavelength *λ*_opt_ minimizes the phase mismatch *Δβ*_QPM_ = 2*β*(*λ*_FH_, *w*) − *β*(*λ*_SH_, *w*):

1 $${\lambda }_{{{{\rm{opt}}}}}(w)=\arg \min \left(\left|2\beta ({\lambda }_{{{{\rm{FH}}}}},w)-\beta ({\lambda }_{{{{\rm{SH}}}}},w)+G\right|\right)$$

where *λ*_SH_ is the second harmonic wavelength, and *λ*_FH_ = 2*λ*_SH_ is the fundamental harmonic wavelength. Ideally *λ*_opt_ would be the intended design wavelength, but due to variation in waveguide top width and the resulting change in geometric dispersion, the optimally phase matched SH wavelength often differs from that which is intended. For a fixed geometry and a given *G* and nominal film thickness *w* = *w*_0_, then, the phase-matching sensitivity is the quantity $\frac{d{\lambda }_{{{{\rm{opt}}}}}}{dw}{| }_{{w}_{0}}$. We approximate this derivative by simulating the interacting modes in the nominal and slightly perturbed geometries. A more detailed description and derivation can be found in the Supplementary Information.

### Theoretical estimation of the conversion efficiency

To obtain a semi-analytical expression for the conversion efficiency in our second harmonic generation experiments, we assume that the fundamental harmonic and second harmonic fields experience only linear losses, no back-conversion from the generated UV back to the pump takes place, and optical powers remain in the undepleted regime. Under these approximations, the conversion efficiency (*η*_abs_) can be written as

2 $${\eta }_{{{{\rm{abs}}}}}:={\left|\frac{{a}_{2}(L)}{{a}_{1}(0)}\right|}^{2}=\frac{4}{{\pi }^{2}}{\left|{\kappa }_{{{{\rm{eff}}}}}\right|}^{2}{\left|{a}_{1}(0)\right|}^{2}{L}^{2}{e}^{-\left({\alpha }_{1}+\frac{{\alpha }_{2}}{2}\right)L}\left(\frac{{\sinh }^{2}\left[\left({\alpha }_{1}-\frac{{\alpha }_{2}}{2}\right)\frac{L}{2}\right]}{{\left[\left({\alpha }_{1}-\frac{{\alpha }_{2}}{2}\right)\frac{L}{2}\right]}^{2}}\right).$$

Here, *a*_1_(0) is the amplitude of the fundamental harmonic field, *a*_2_(*L*) is the amplitude of the second harmonic (UV) field at the waveguide output *z* = *L*, *κ*_eff_ is the effective coupling constant proportional to the second-order susceptibility *d*_*zzz*_, *α*_1_ and *α*_2_ are the measured linear propagation losses for the fundamental and second harmonic fields, respectively, and *L* is the length of the poled waveguide. The exponential factor accounts for amplitude attenuation due to losses, and the hyperbolic sine term captures the interplay between pump and second harmonic attenuation rates. More details can be found in the Supplementary Information.

### Ordinary differential equation (ODE) model

The coupled differential equations used to model frequency conversion from the fundamental field *a*_1_ to the second harmonic field *a*_2_ as a function of propagation length *z* incorporating linear and nonlinear losses are

3 $$\frac{d{a}_{1}}{dz}=-i\kappa {a}_{2}{a}_{1}^{*}{e}^{i\Delta \beta z}-\frac{{\alpha }_{1}}{2}{a}_{1}-\frac{1}{2}\left(\frac{{\beta }_{11}}{{A}_{{{{\rm{eff}}}},1}}{\left|{a}_{1}\right|}^{2}+\frac{{\beta }_{12}}{{A}_{{{{\rm{eff}}}},{{{\rm{mix}}}}}}{\left|{a}_{2}\right|}^{2}\right){a}_{1}$$

4 $$\frac{d{a}_{2}}{dz}=-i\kappa {\left|{a}_{1}\right|}^{2}{e}^{-i\Delta \beta z}-\frac{{\alpha }_{2}}{2}{a}_{2}-\frac{1}{2}\left(\frac{{\beta }_{21}}{{A}_{{{{\rm{eff}}}},{{{\rm{mix}}}}}}{\left|{a}_{1}\right|}^{2}+\frac{{\beta }_{22}}{{A}_{{{{\rm{eff}}}},2}}{\left|{a}_{2}\right|}^{2}\right){a}_{2}$$

where *κ* is the second harmonic generation coupling, *Δβ* is the phase mismatch, *α*_*i*_ are the measured linear absorption coefficients, *β*_*ij*_ are the two photon absorption coefficients for photons from *a*_*i*_ and *a*_*j*_, *A*_eff,j_ are the effective mode areas of the fields, and *A*_eff,mix_ is the average of the effective mode areas of *a*_1_ and *a*_2_.

These equations are solved numerically to determine *a*_2_ as a function of input fundamental power. The parameters are allowed to vary within experimental error in order to fit the experimental data. More details can be found in the Supplementary Information.

## Supplementary information

Supplementary Information

Transparent Peer Review file

## Data Availability

Data supporting the plots and other findings are available from the corresponding author upon request.
