# Supplementary material for: Milliwatt-level UV generation using sidewall poled lithium niobate
Source: Nat Commun. 2026 Apr 21;17:3651. doi: 10.1038/s41467-026-68524-y (PMC13100000; doi:10.1038/s41467-026-68524-y)
Supplement: Supplementary file 1 — Supplementary Information [file 41467_2026_68524_MOESM1_ESM.pdf]

Milliwatt-level UV generation using sidewall poled lithium niobate

C.A.A. Franken[1,2,3,*], S.S. Ghosh[1,4,5,*], C.C. Rodrigues[1,6], J. Yang[1], C.J. Xin[1], S. Lu[1], D. Witt[1], G. Joe[1], G.S. Wiederhecker[6], K.-J. Boller[2], and M. Lončar[1,†]

[1] *School of Engineering and Applied Sciences, Harvard University, Cambridge, Massachusetts, United States of America*
[2] *Laser Physics and Nonlinear Optics Group, Department of Science and Technology, MESA+ Institute of Nanotechnology, University of Twente, Enschede, Overijssel, the Netherlands*
[3] *Sabratha Photonics B.V., Enschede, Overijssel, the Netherlands*
[4] *Department of Physics, Harvard University, Cambridge, Massachusetts, United States of America*
[5] *Fermi National Accelerator Laboratory, Batavia, Illinois, United States of America*
[6] *Gleb Wataghin Physics Institute, University of Campinas, Campinas, São Paulo, Brazil*
[*] *These authors contributed equally to this work.*
[†] *Electronic mail: loncar@seas.harvard.edu*

Supplementary Information

Supplementary Note 1. Phase matching sensitivity calculation

The phase matching sensitivity is defined as the derivative of the optimally phase matched second harmonic (SH) wavelength (in nm) with respect to waveguide top width (also measured in nm). For a given SH wavelength λ_{SH}, the second harmonic generation phase mismatch is given as $\Delta\beta(\lambda_{\mathrm{SH}}, w, ...) = 2\beta(\lambda_{\mathrm{FH}}, w, ...) - \beta(\lambda_{\mathrm{SH}}, w, ...)$, where $\lambda_{\mathrm{FH}} = 2\lambda_{\mathrm{SH}}$ and the mode propagation constants β are determined by both the material dispersion of the crystal and the geometric dispersion of the waveguide. The geometry of the waveguide is mainly parametrized by the waveguide top width w, film thickness, etch depth and sidewall angle. In this treatment we focus on the effect of the top width w specifically. The presence of a constant poling period Λ contributes a fixed quasi-phase matched grating momentum $G = 2\pi/\Lambda$, which we then write overall as $\Delta\beta_{\mathrm{QPM}}(\lambda_{\mathrm{SH}}, w) = 2\beta(\lambda_{\mathrm{FH}}, w) - \beta(\lambda_{\mathrm{SH}}, w) + G$. For a given G and w the phase matched SHG wavelength (λ_{opt}) is the one that minimizes $\Delta\beta_{\mathrm{QPM}}$:

$$\lambda_{\mathrm{opt}}(w) = \arg\min\left(|2\beta(\lambda_{\mathrm{FH}}, w) - \beta(\lambda_{\mathrm{SH}}, w) + G|\right) \tag{1}$$

However, due to waveguide width nonuniformity, which impacts the geometric dispersion, the optimally phase matched second harmonic wavelength often differs from this value. For an otherwise fixed geometry and a given G and nominal waveguide width $w = w_0$, then, the phase matching sensitivity is defined as $\left.\frac{d\lambda_{\mathrm{opt}}}{dw}\right|_{w_0}$. To evaluate this sensitivity numerical modeling is used to calculate λ_{opt} for different waveguide top widths, assuming particular values for film thickness, etch depth, and quasi-phase matched grating momentum G. Even though film thickness and etch depth variations can contribute to sensitivity, they are not considered here since these variations are accounted for by the adapted poling method used. However, an earlier study [1], confirmed by our own calculations in the text below, concluded that thick and wide waveguides have the lowest sensitivity to film thickness variations.

The derivative is calculated numerically. Specifically, we calculate $(w_-, \lambda_{\mathrm{opt}}^-)$, $(w_0, \lambda_{\mathrm{opt}}^0)$, and $(w_+, \lambda_{\mathrm{opt}}^+)$, where $w_\mp = w_0 \mp \delta w$ are small perturbations from the nominal waveguide width w_0 and $\lambda_{\mathrm{opt}}^\mp$ are the corresponding values for phase matched SHG wavelength. The slope of the least mean squares linear fit between these three points is then taken as an approximation to $\left.\frac{d\lambda_{\mathrm{opt}}}{dw}\right|_{w_0}$.

Similarly, the phase matching sensitivity is also evaluated for variations in film thickness. For a given film thickness $t = t_0$ the sensitivity is defined as $\left.\frac{d\lambda_{\mathrm{opt}}}{dt}\right|_{t_0}$ and evaluated for a range of waveguide top widths (Supplementary Fig. 1). Like in Fig. 2a in the main text, we find that wider waveguides have a lower phase matching sensitivity to fabrication tolerances (in this case to film thickness variations).

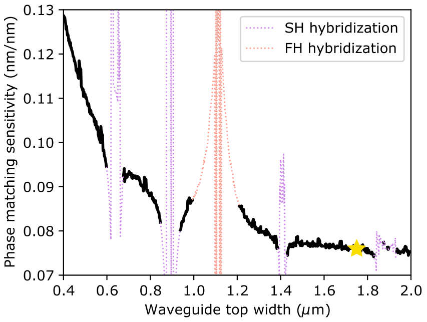

Supplementary Figure 1: Phase matching sensitivity with respect to film thickness variation, for different waveguide top widths. The star denotes the chosen waveguide width of 1.75 μm

Supplementary Note 2. Waveguide propagation loss and fiber-to-chip coupling loss

The propagation loss and fiber-to-chip coupling loss for the fiber-to-chip coupling is measured by fabricating a set of spiral waveguides of varying length and measuring the transmission at the wavelengths of interest. The set-up is described in the Methods of the main text. The fibers used here are PM-630HP fibers (OZ Optics). For transmission measurements at 780 nm a CW Toptica DL Pro 780 laser is used. For transmission measurements at 405 nm a CW, fiber-pigtailed, Fabry-Pérot laser diode is used (QPhotonics QFLD-405-30SAX-PM) in combination with a laser diode driver (Thorlabs CLD1010LP). The power is measured using a calibrated photodiode (Thorlabs S150C). To interpret our measurements we assume a symmetric fiber-to-chip coupling loss. We support this assumption by verifying that the incident beam from the lensed fiber is single transverse mode for both wavelengths.

The transmission as a function of propagation length through the spiral structures, for both wavelengths, is shown in Supplementary Fig. 2. Measurements of spirals with defects, as can also be seen as bright scatter points in the images in Supplementary Fig. 3 to 6, are excluded from the linear fit that estimates the propagation and fiber-to-chip coupling loss. As the facets of both chips are the same, the average coupling loss can be determined from spirals of both waveguide widths. We find an average fiber-to-chip coupling loss of 9.32 ± 0.48 dB and 4.77 ± 0.37 dB for 405 nm and 780 nm, respectively.

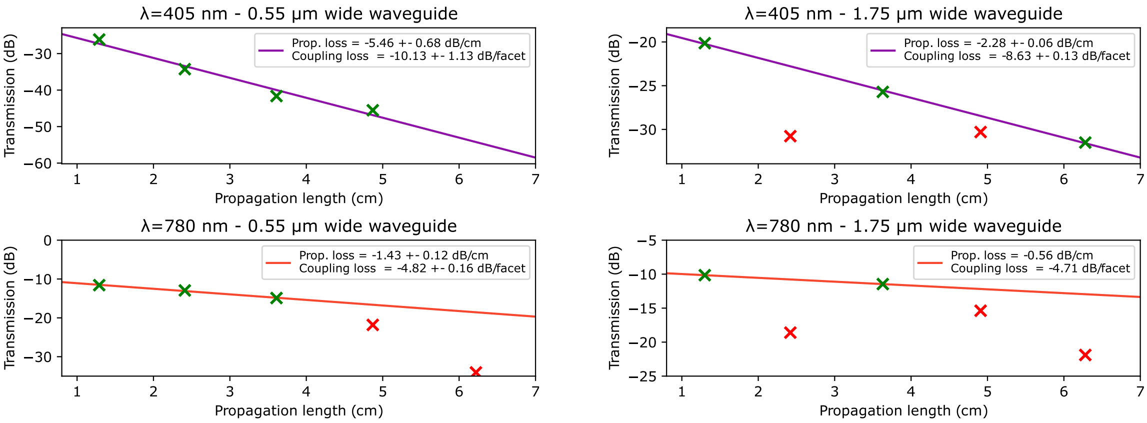

Supplementary Figure 2: Transmission measurements for waveguide spirals of various lengths with two waveguide top widths, 0.55 and 1.75 μm, measured at two wavelengths, 405 and 780 nm. The red markers indicate spiral measurements where the transmission measurement is not accurate due to a fabrication error in the spiral structure (see also Supplementary Fig. 3 to 6). Only the green marked measurements are used for the linear fit and loss extraction.

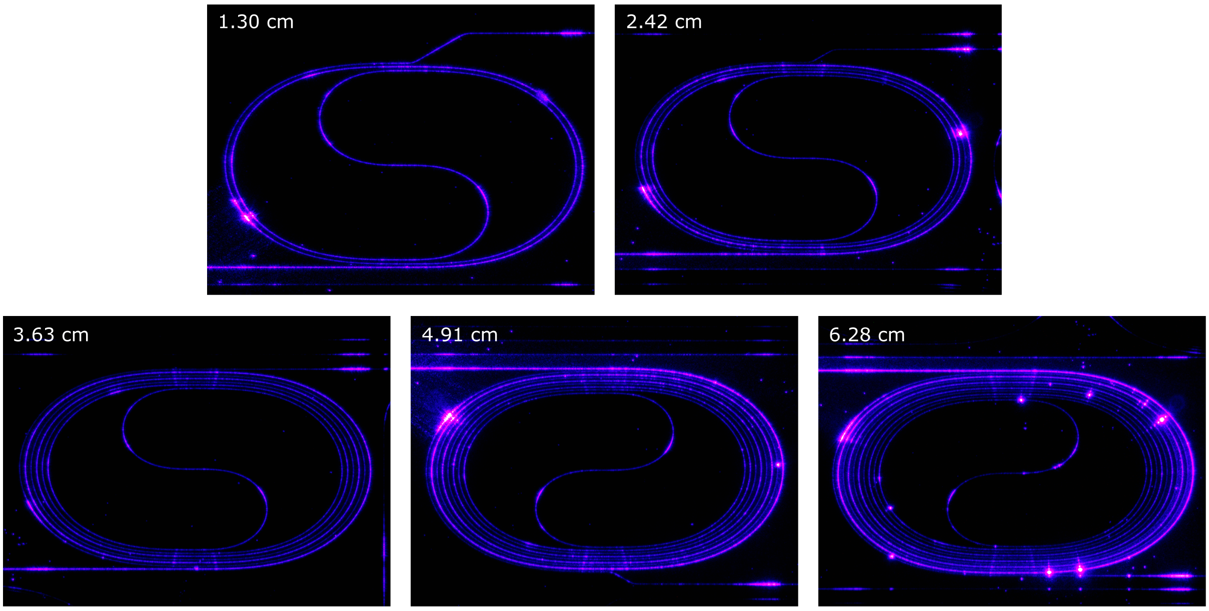

Supplementary Figure 3: Top-down microscope images of 1.75 μm wide waveguide spirals with 405 nm light at the input.

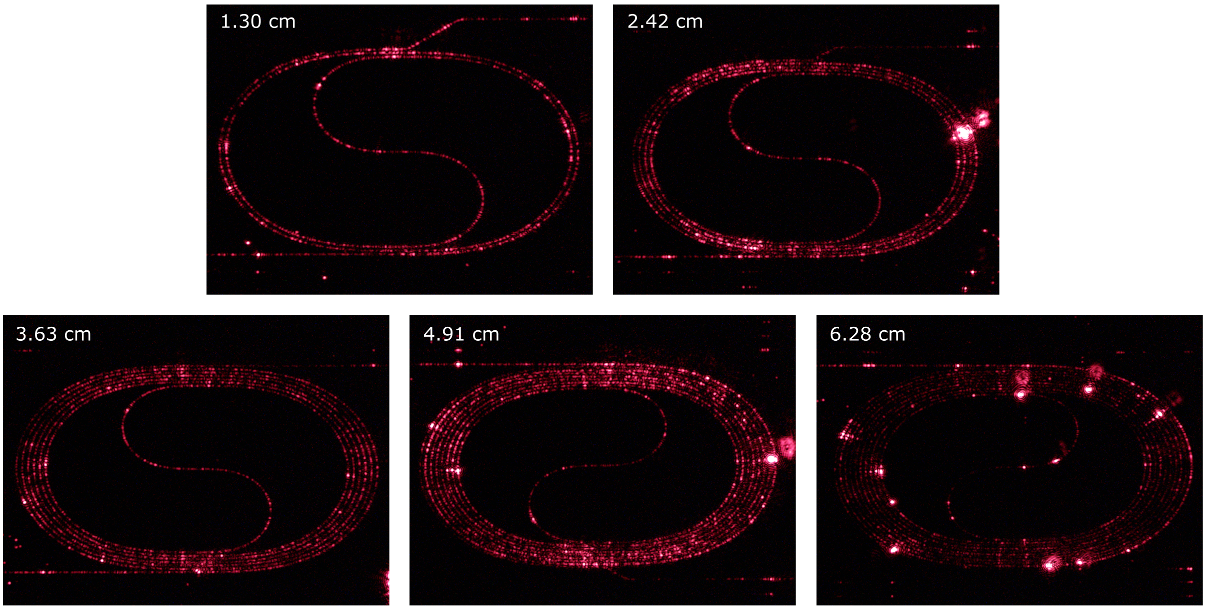

Supplementary Figure 4: Top-down microscope images of 1.75 μm wide waveguide spirals with 780 nm light at the input.

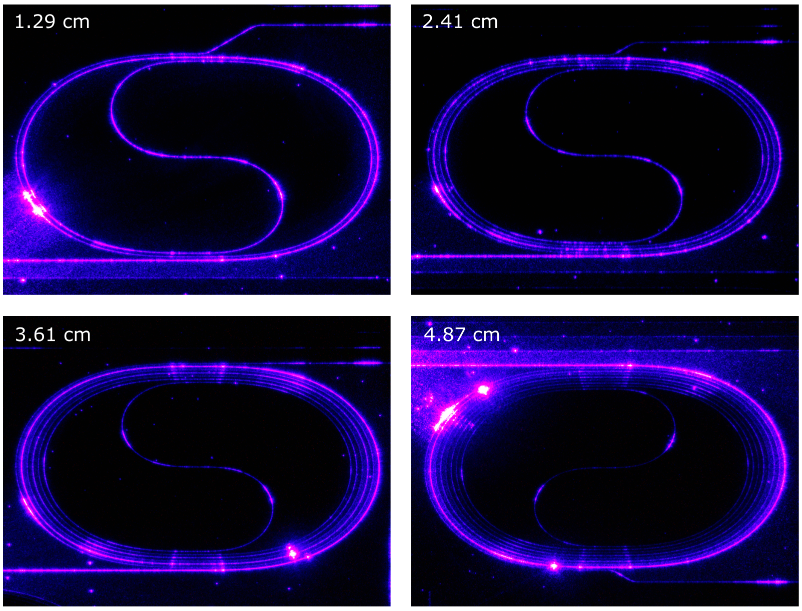

Supplementary Figure 5: Top-down microscope images of 0.55 μm wide waveguide spirals with 405 nm light at the input. The fifth spiral of 6.22 cm did not transmit any measurable 405 nm light, no image was taken for this spiral.

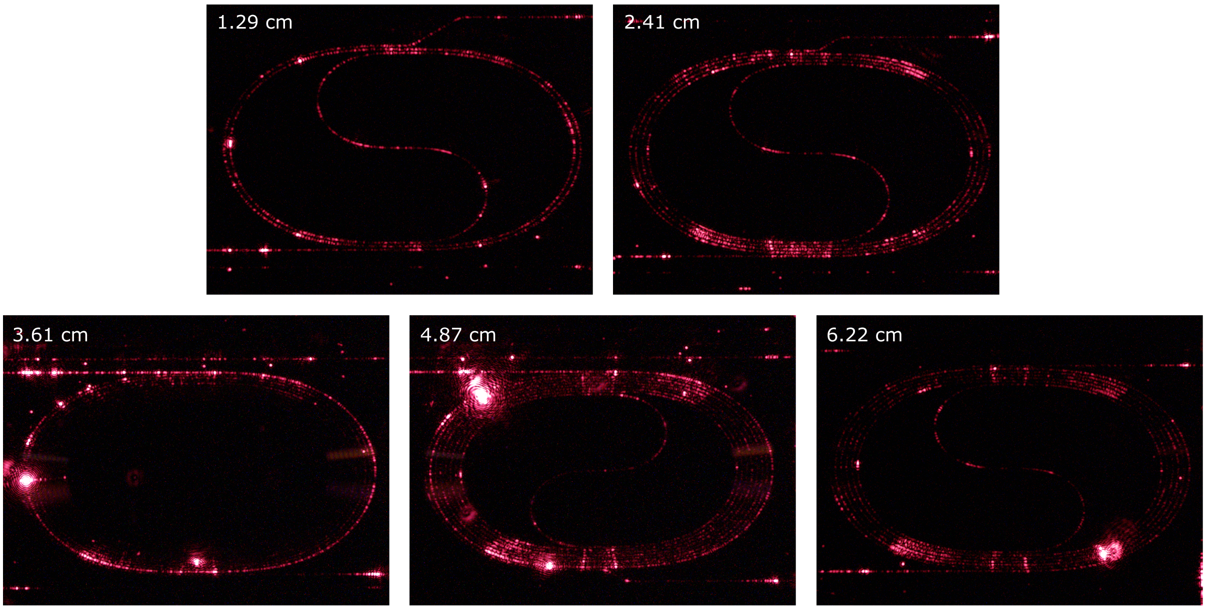

Supplementary Figure 6: Top-down microscope images of 0.55 μm wide waveguide spirals with 780 nm light at the input.

Supplementary Note 3. Film thickness and etch depth variation

In our adapted poling process the film thickness and etch depth are taken into account to calculate the optimal poling period along the waveguide. The film is measured, before and after etching, using a Woollam RC2 Spectroscopic Ellipsometer. The horizontal spacing between data points is 100 μm, measured at the center of each waveguide. The results are shown in Supplementary Fig. 7.

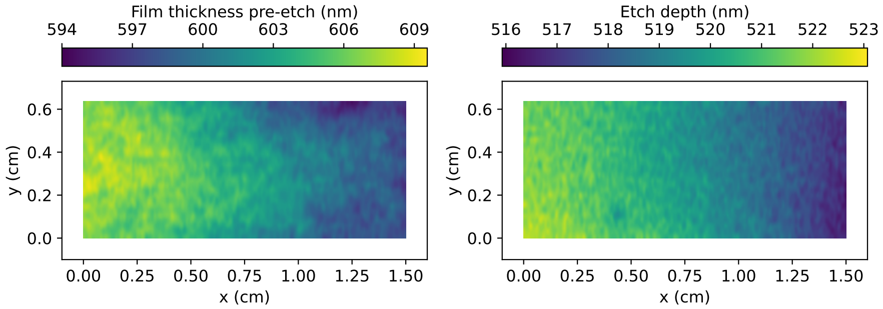

Supplementary Figure 7: Left, lithium niobate film thickness before etching the waveguides. Right, etch depth measured after waveguide definition. Here, the etch depth is the film thickness after etching subtracted from the film thickness before etching.

Supplementary Note 4. Optical intensity at the waveguide sidewall

Several factors contribute to the total waveguide propagation loss, such as linear absorption, non-linear (two-photon) absorption, and sidewall scattering. The last factor is highly dependent on the waveguide geometry and mode confinement, since the intensity of the optical mode on the sidewall is directly proportional to the scattering loss. Scattering loss from sidewall roughness is proportional to $1/\lambda^3$ [2], therefore we will only consider the dominant scattering loss from the second harmonic UV mode here. To investigate what waveguide geometry is optimal, the sidewall intensity for the second harmonic (SH) mode is calculated for various waveguide widths using COMSOL (Supplementary Fig. 8a and b). At some waveguide widths the fundamental mode has a TE polarization below 90%, these regions are labeled as regions with hybridized modes and indicated with a dotted line. The trend of the solid line shows that wider waveguides have lower sidewall intensity, thus lower sidewall scattering loss.

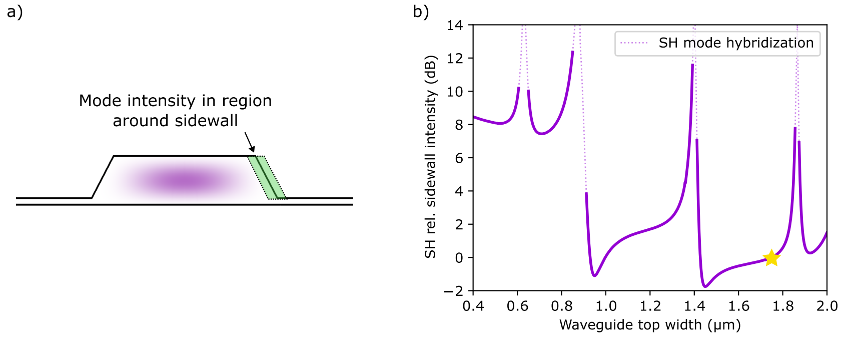

Supplementary Figure 8: a) During the simulation the mode intensity is calculated in an area surrounding the waveguide sidewall. b) Relative sidewall intensity of the second harmonic UV mode as a function of waveguide width. The intensity is related to the intensity at our waveguide width of 1.75 μm, indicated by the star.

Supplementary Note 5. Data analysis to obtain the duty cycle from SH microscope images

A high-resolution second harmonic microscope with an oil-immersion objective is used to determine the duty cycle of our poled structures, as described in the main text. For chip #1 and #2 we have recorded 25 and 26 second harmonic microscope images, respectively. A single data point sacrifices a single test structure, since these can be poled reliably only once. For the results shown in the main text (Fig. 2c) a total of 51 test structures were used, each about 500 µm in length with a poling period that is comparable to what is used for the 1.5 cm long, functional SPLN waveguides. The analysis to obtain the duty cycle from a single device is described in the caption of Supplementary Fig. 9.

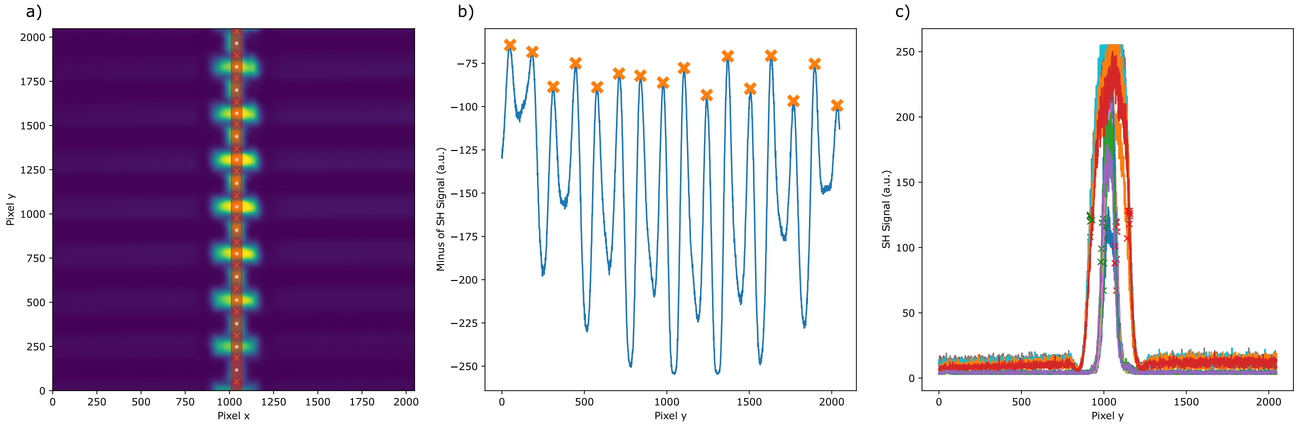

Supplementary Figure 9: a) Second harmonic microscope image, here the SH signal, of a sidewall poled lithium niobate waveguide with the poling fingers still on the waveguide. The red shaded area denotes what part of the image will be used to determine the duty cycle. b) The data in the red shaded area (see a) averaged along x multiplied by -1. Peaks in this line (orange cross-markers) correspond to dark points/lines in the SH microscope image, which indicate a boundary of the poled domains in lithium niobate. c) Cross-sections of the image (along x) at the white dots in a). This allows us to determine whether a domain is inverted or not inverted by comparing the widths of the curves. The inverted domains are partially obscured by the poling fingers which reduces their width in these cross-sectional plots. Using this we can determine which part of the image is inverted and whether the duty cycle is below or above 50%. The duty cycle reported is the mean duty cycle extracted and the error is the standard deviation of the duty cycle extracted from the image through the peak finding algorithm. The extracted duty cycle for this image is 51.39 ± 0.96 % for a poling voltage of 139 V.

Supplementary Note 6. Calibration of the phase matching function

The experimental setup and measurement procedure used to obtain the phase matching function are described in detail in the Methods section of the main text. Here, we focus on how the raw photodiode data is processed to produce the phase matching curve shown in Fig. 3b of the main text. Each step of this procedure is illustrated in Fig. 10:

(a) **Raw photodiode trace and calibration.** As the fundamental harmonic (FH) pump laser is swept in wavelength across the phase matching region, the output of the UV SPLN waveguide is measured on a UV photodiode (with a pump filter). The photodiode voltage is recorded as a function of the FH wavelength. After the sweep, at the peak-conversion wavelength, the UV power is measured with a calibrated power meter. This single calibration point—mapping photodiode voltage (V) to optical power (mW)—is then applied to convert the entire photodiode trace into UV power. In the example shown, the on-chip UV power at peak efficiency is 2.7 mW (corresponding to 1.60 V on the photodiode), indicated by the black cross in subfigure (a). This occurs at an on-chip pump power of 8.90 mW and an FH wavelength of 778.87 nm.

(b) **Pump power–based data selection.** A 99/1 % fiber splitter is placed at the pump laser output, sending 1 % of the FH light to a wavelength meter that also measures input power. Because the laser power fluctuates during the sweep, only data points whose pump power lies within ±10 % of the reference power measured at the calibration point (the black cross) are retained.

(c) **Selected data pump power vs. wavelength.** After applying this ±10 % pump power selection, the remaining on-chip FH pump power is plotted as a function of the FH wavelength.

(d) **Selected data UV power vs. wavelength.** The corresponding UV powers, for the same selected data points, are plotted versus FH wavelength.

(e) **Binning and averaging.** To reduce noise from alignment drifts (likely caused by small fiber vibrations), the data are grouped into 4 pm wavelength bins and averaged (the solid line). The shaded region indicates the minimum-to-maximum spread within each bin. Some bins contain no data points (due to the ±10 % filtering) but lie outside the main phase-matched region (e.g., at 779.2 nm), so they do not affect the overall shape of the phase matching function.

(f) **Offset drift correction.** Over the 3–4 min duration of the measurement, a small DC-voltage drift in the photodiode (on the order of 10 mV) could lead to negative power readings. To correct for this, the photodiode noise floor is measured at the start and end of the sweep, and its slope is subtracted from the dataset so that the noise floor remains near zero throughout.

(g) **Final comparison with theory.** The fully processed data are plotted alongside results from the coupled ordinary differential equation (ODE) model. The measured on-chip pump power and wavelength serve as inputs to the model; the band around the theoretical curve shows the effect of the ±10 % pump-power variation on the UV output power.

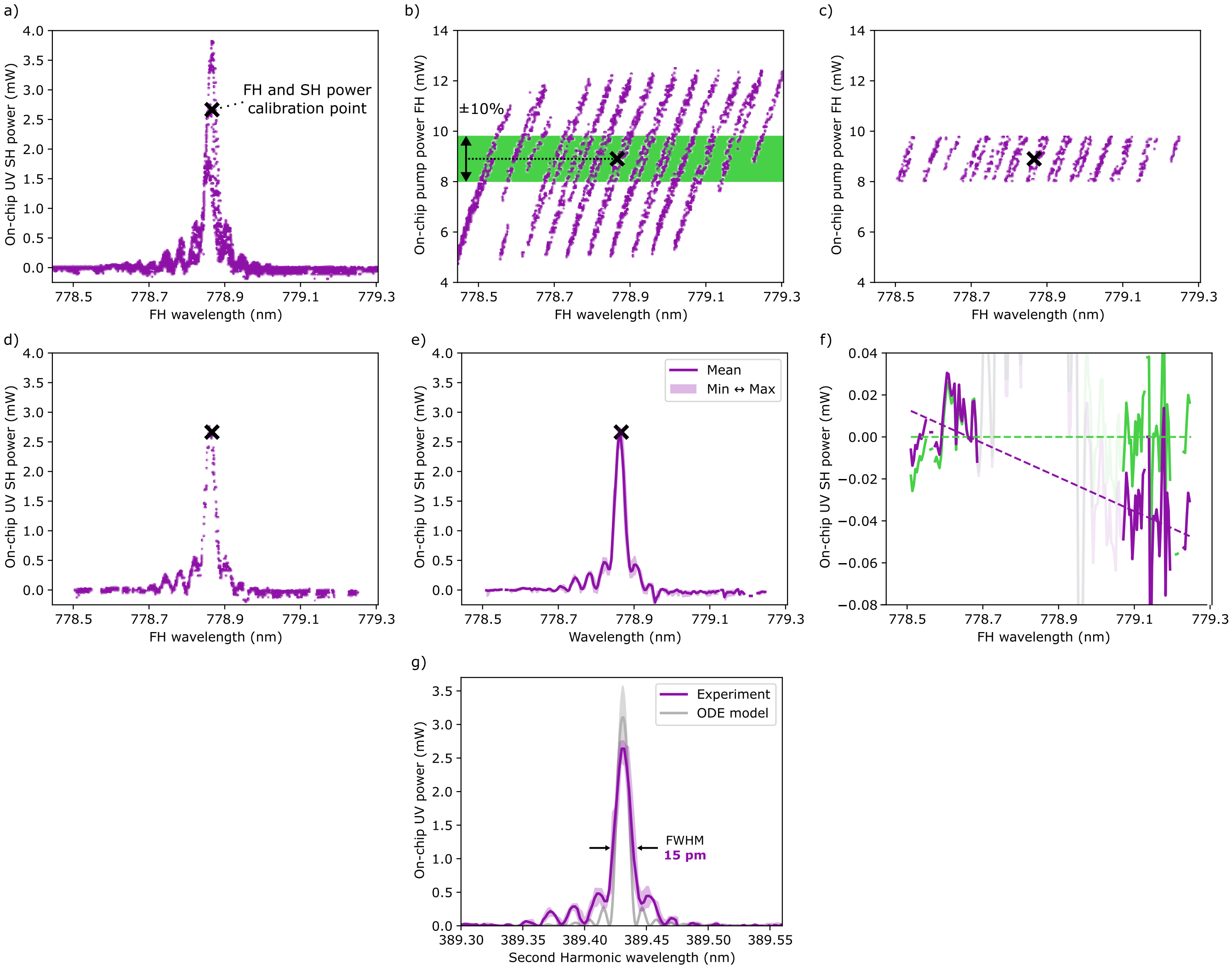

Supplementary Figure 10: Data analysis to obtain the phase matching function from the raw experimental data. See text in this section for a subfigure description.

Supplementary Note 7. Theoretical estimation of the normalized conversion efficiency

We model second harmonic generation (SHG) using the following dynamic equations that describe the propagation of the waves along the waveguide oriented in the z direction (aligned with the Y material axis).

$$\frac{da_1}{dz} = -i\kappa^* a_1^* a_2 e^{i\Delta\beta z} - \frac{\alpha_1}{2} a_1 \quad \text{and} \quad \frac{da_2}{dz} = -i\kappa a_1^2 e^{-i\Delta\beta z} - \frac{\alpha_2}{2} a_2. \tag{2}$$

The functions $a_1(z)$ and $a_2(z)$ are the electric field intensities for the fundamental and second harmonic fields, respectively. The parameter $\Delta\beta = \beta_2 - 2\beta_1$ is the phase mismatch between the wavevectors β_1 and β_2; α_1 and α_2 are the linear propagation losses; and κ is the coupling constant for the SHG interaction. The equations are symmetrically coupled when normalized to intensity due to the degeneracy of the upconversion process $2\omega = \omega + \omega$ and the non-degeneracy of the simultaneous downconversion process $\omega = 2\omega - \omega$. This system does not have an analytic solution. Therefore, to obtain a semi-analytical expression to guide our intuition for the conversion efficiency, we assume that the pump intensity (a_1) is independent of the SHG interaction. That is, the pump only experiences linear losses, and there is no back-conversion from the second harmonic to the fundamental harmonic. Thus

$$\frac{da_1}{dz} \approx -\frac{\alpha_1}{2} a_1 \quad \Rightarrow \quad a_1(z) = a_1(0) e^{-\frac{\alpha_1}{2} z} \tag{3}$$

the evolution of the second harmonic is then given by

$$\frac{da_2}{dz} \approx -\frac{\alpha_2}{2} a_2 - i\kappa \left(a_1(0) e^{-\frac{\alpha_1}{2} z} \right)^2 e^{-i\Delta\beta z}. \tag{4}$$

The idea of periodic poling is precisely to correct the phase mismatch represented by the exponential $e^{-i\Delta\beta z}$ in Eq. 2. To eliminate this term, we modulate κ in such a way that $\kappa \sim e^{i\Delta\beta z}$. κ is proportional to the second-order nonlinear susceptibility d_{ijk} through the equation

$$\kappa = \frac{\varepsilon_0 \omega_{\mathrm{SH}}}{v_{g,\mathrm{SH}}} \left(\int_S \varepsilon_{ij} E_2^i E_2^j dS \right)^{-1} \int_S d_{ijk} E_2^i E_1^j E_1^k dS, \tag{5}$$

where E_1^i is the i-th component of the fundamental harmonic; E_2^i is the i-th component of the second harmonic; ε_0 is the vacuum electric permittivity; ε_{ij} is the (i, j)-th component of the electric permittivity tensor; $v_{g,\mathrm{SH}}$ is the group velocity of the second harmonic field and ω_{SH} is the angular frequency of the second harmonic field. We can now directly modulate d_{iJ} (using contracted Voigt notation for the indices) in order to achieve phase matching as follows

$$d_{iJ}(z) \approx \begin{pmatrix} 0 & 0 & 0 & 0 & 0 & 0 \\ 0 & 0 & 0 & 0 & 0 & 0 \\ 0 & 0 & d_{33} & 0 & 0 & 0 \end{pmatrix} \mathrm{sign}\left[\cos\left(\Delta\beta z\right)\right] = d_{zzz} \mathrm{sign}\left[\cos\left(\Delta\beta z\right)\right] \delta_{iz}\delta_{jz}\delta_{kz} \tag{6}$$

where the $\delta_{iz}, \delta_{jz}, \delta_{kz}$ are Kronecker delta functions.

Here, since most of the SHG comes from the $d_{33} = d_{zzz}$ coefficient, we assumed that only this coefficient is non-zero. The choice of a square wave modulation is supported by our experimental data that indicate that our domains are square-shaped. Then, the coupling parameter κ becomes

$$\kappa = \underbrace{\left(\frac{\varepsilon_0 \omega_{\mathrm{SH}}}{v_{g,\mathrm{SH}}} \frac{\int_S d_{zzz} E_2^z E_1^z E_1^z dS}{\int_S \varepsilon_{ij} E_2^i E_2^j dS} \right)}_{:=\kappa_{\mathrm{eff}}} \mathrm{sign}\left[\cos\left(\Delta\beta z\right)\right] := \kappa_{\mathrm{eff}}\, \mathrm{sign}\left[\cos\left(\Delta\beta z\right)\right], \tag{7}$$

The equation we need to solve is then given by

$$\frac{da_2}{dz} \approx -\frac{\alpha_2}{2}a_2 - i\kappa_{\text{eff}}\,\text{sign}\left[\cos\left(\Delta\beta z\right)\right]\left[a_1(0)\right]^2 e^{-\alpha_1 z}e^{-i\Delta\beta z}. \tag{8}$$

The next step is to expand the function $\text{sign}\left[\cos\left(\Delta\beta z\right)\right]$ into its Fourier series, which is given by

$$\text{sign}\left[\cos\left(\Delta\beta z\right)\right] = \frac{4}{\pi}\sum_{n=0}^{\infty}\left(-1\right)^n \frac{\cos\left[\left(2n+1\right)\Delta\beta z\right]}{2n+1} \approx \frac{4}{\pi}\cos\left(\Delta\beta z\right). \tag{9}$$

Substituting the function with only the first term of its Fourier series, rewriting and averaging out the fast term, we then have

$$\begin{aligned}
\frac{da_2}{dz} &\approx -\frac{\alpha_2}{2}a_2 - i\frac{4}{\pi}\kappa_{\text{eff}}[a_1(0)]^2 e^{-\alpha_1 z}\cos(\Delta\beta z)e^{-i\Delta\beta z} \\
&= -\frac{\alpha_2}{2}a_2 - i\frac{4}{\pi}\kappa_{\text{eff}}[a_1(0)]^2 e^{-\alpha_1 z}\frac{1+e^{-2i\Delta\beta z}}{2} \\
&\approx -\frac{\alpha_2}{2}a_2 - i\frac{4}{\pi}\kappa_{\text{eff}}[a_1(0)]^2 e^{-\alpha_1 z}\cdot\frac{1}{2} \quad \text{(averaging out } e^{-2i\Delta\beta z}\text{)} \\
&= -\frac{\alpha_2}{2}a_2 - i\frac{2}{\pi}\kappa_{\text{eff}}[a_1(0)]^2 e^{-\alpha_1 z}.
\end{aligned} \tag{10}$$

and this equation has an analytical solution given by

$$a_2(z) = i\frac{2}{\pi}\kappa_{\text{eff}}\left|a_1(0)\right|^2 e^{-\frac{\alpha_2}{2}z}\left(\frac{1-e^{\left(\frac{\alpha_2}{2}-\alpha_1\right)z}}{\frac{\alpha_2}{2}-\alpha_1}\right) \tag{11}$$

Then, the absolute conversion efficiency can be written as:

$$\eta_{\text{abs}} := \left|\frac{a_2(L)}{a_1(0)}\right|^2 = \frac{4}{\pi^2}\left|\kappa_{\text{eff}}\right|^2\left|a_1(0)\right|^2 L^2 e^{-\left(\alpha_1+\frac{\alpha_2}{2}\right)L}\left(\frac{\sinh^2\left[\left(\alpha_1-\frac{\alpha_2}{2}\right)\frac{L}{2}\right]}{\left[\left(\alpha_1-\frac{\alpha_2}{2}\right)\frac{L}{2}\right]^2}\right). \tag{12}$$

Even though this derivation assumed only the d_{zzz} coefficient, we emphasize that in the COMSOL simulation, all coefficients were considered non-zero and a full vectorial simulation was performed. Normalizing the fields such that the incident pump power is $\left|a_1(0)\right|^2 = 1$ [W], the numerical efficiency value was found to be

$$\frac{\eta_{\text{abs}}}{\left|a_1(0)\right|^2 L^2} = 9048\left[\frac{\%}{\text{W.cm}^2}\right] \tag{13}$$

in which we used $d_{33} = 25$ [pm/V], $d_{31} = 4.9$[pm/V] and $d_{22} = 2.2$[pm/V] at 780 [nm]. The geometry parameters were $L = 1.5$ [cm], $\alpha_1 = 12.3$ [1/m], $\alpha_2 = 53.0$ [1/m], waveguide top width 1750 [nm], LN thickness 600 [nm], sidewall angle 62.5° and slab thickness 80 [nm] on top of a silicon dioxide substrate. We note that if we considered only d_{33} to be non-zero, the efficiency drops to 9047 [%/W.cm^2]. This is only 1[%/W.cm^2] smaller than the efficiency predicted using all components of the d_{iJ} tensor, proving that indeed it is sufficient to consider only d_{33} - the leading coefficient in the conversion. If we set the pump to be lossless ($\alpha_1 = 0$) then we get an efficiency of 11084 [%/W.cm^2]. This value increases to 16275 [%/W.cm^2] if both fields are lossless ($\alpha_1 = \alpha_2 = 0$). This supports the necessity of including linear propagation losses to properly estimate the conversion efficiency.

Supplementary Note 8. Ordinary differential equation (ODE) model

The coupled ordinary differential equations for the fundamental harmonic amplitude a_1 and second harmonic field amplitude a_2 accounting for second-harmonic generation and linear absorption are given by

$$\frac{da_1}{dz} = -i\kappa a_2 a_1^* e^{i\Delta\beta z} - \frac{\alpha_1}{2} a_1 \tag{14}$$

$$\frac{da_2}{dz} = -i\kappa a_1^2 e^{-i\Delta\beta z} - \frac{\alpha_2}{2} a_2 \tag{15}$$

where κ is the second-harmonic generation coupling between a_1 and a_2, z is the coordinate in the propagation direction, and $\Delta\beta$ is the phase mismatch. The equations are symmetrically coupled when normalized to intensity due to the degeneracy of the upconversion process $2\omega = \omega + \omega$ and the non-degeneracy of the simultaneous downconversion process $\omega = 2\omega - \omega$. Here, κ can be derived from the experimentally obtained power and length normalized conversion efficiency η with units [$\%\mathrm{W}^{-1}\mathrm{cm}^{-2}$] as $\kappa = \sqrt{\eta}$. The experimentally obtained values of α_i are measured as a function of intensity, hence the factor $\frac{1}{2}$ for the field amplitude attenuation in Eqs. 14 and 15. More details about our spiral loss measurements can be found in the main text and a previous section of the Supplementary Information.

So far, this model does not account for two-photon absorption (TPA), an intensity-dependent loss. The differential equation describing *only* TPA is

$$\frac{dI}{dz} = -\beta_{TPA} I^2 \tag{16}$$

where β_{TPA} is the TPA coupling coefficient and I is the intensity of the field [3]. Recasting this equation in terms of field amplitudes $a = \sqrt{I A_{\text{eff}}}$, where A_{eff} is the effective area of the mode:

$$\frac{d}{dz}\frac{|a|^2}{A_{\text{eff}}} = -\beta_{TPA}\frac{|a|^4}{A_{\text{eff}}^2} \tag{17}$$

which in terms of $d|a|/dz$ is

$$\frac{d\,|a|}{dz} = \frac{-\beta_{TPA}}{2A_{\text{eff}}}\,|a|^2\,a \tag{18}$$

This is the form of the TPA coupling term for a given field a. The TPA components of the differential equations for our two fields a_1 and a_2 are then

$$\frac{da_1}{dz} = -\frac{1}{2}\left(\frac{\beta_{12}}{A_{\text{eff,mix}}}|a_2|^2 + \frac{\beta_{11}}{A_{\text{eff},1}}|a_1|^2\right)a_1 \tag{19}$$

$$\frac{da_2}{dz} = -\frac{1}{2}\left(\frac{\beta_{21}}{A_{\text{eff,mix}}}|a_1|^2 + \frac{\beta_{22}}{A_{\text{eff},2}}|a_2|^2\right)a_2 \tag{20}$$

where β_{ij} is the two photon absorption coefficient for photons from a_i and a_j and $A_{\text{eff,mix}}$ is the average of the effective mode areas of a_1 and a_2. The energy of two second harmonic 390 nm photons exceeds the band gap of lithium niobate. So does the energy of one fundamental harmonic 780 nm photon and one second harmonic 390 nm photon - thus the cross terms β_{12} and β_{21}.

Therefore, the complete differential equations used to model frequency conversion and linear and nonlinear losses are

$$\frac{da_1}{dz} = -i\kappa a_2 a_1^* e^{i\Delta\beta z} - \frac{\alpha_1}{2}a_1 - \frac{1}{2}\left(\frac{\beta_{11}}{A_{\text{eff},1}}|a_1|^2 + \frac{\beta_{12}}{A_{\text{eff,mix}}}|a_2|^2\right)a_1 \tag{21}$$

$$\frac{da_2}{dz} = -i\kappa |a_1|^2 e^{-i\Delta\beta z} - \frac{\alpha_2}{2} a_2 - \frac{1}{2}\left(\frac{\beta_{21}}{A_{\text{eff,mix}}} |a_1|^2 + \frac{\beta_{22}}{A_{\text{eff,2}}} |a_2|^2\right) a_2 \tag{22}$$

A comparison of our experimental results and the theoretical model described here is shown in Supplementary Fig. 11. Here, model 1 refers to the model parameters based on literature values [3], [4], as described in the main text. The parameters are allowed to vary within the uncertainties reported in their respective references. Model 2 refers to a free fit of all TPA parameters. For both models the parameters are shown in Supplementary Table 1 and 2. The fitting parameters α_1 and α_2 are only fit in the bounds of their experimental errors in both models. The parameters β_{11}, β_{12}, β_{21} and β_{22} are fit freely in model 2. The initial value for κ is set by calculating its value from the undepleted regime of the measured data; it is then allowed to vary $\pm 50\%$ in both models. A fit condition $\beta_{12} = \beta_{21}$ is added for both models.

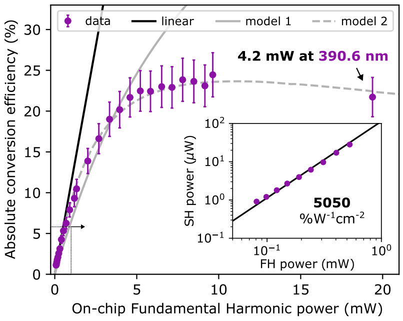

Supplementary Figure 11: Experimental data and results from the numerical model described in this section. Model 1 and 2 represent the same model solved with different parameters, as described in Supplementary Table 1 and 2, respectively.

Supplementary Table 1: Model 1 parameter values

Name	Value	Allowed to vary?
κ	7.662 $W^{-1/2}cm^{-1}$	$\pm 50\%$
α_1	0.1566 cm^{-1}	Within uncertainty
α_2	0.5412 cm^{-1}	Within uncertainty
β_{11}	0 cm/W	No
β_{12}	1.012e-09 cm/W	Within uncertainty
β_{21}	1.012e-09 cm/W	Within uncertainty
β_{22}	4.500e-09 cm/W	Within uncertainty

Supplementary Table 2: Model 2 parameter values

Name	Value	Allowed to vary?
κ	9.463 $W^{-1/2}cm^{-1}$	$\pm 50\%$
α_1	0.1037 cm^{-1}	Within uncertainty
α_2	0.5200 cm^{-1}	Within uncertainty
β_{11}	8.105e-07 cm/W	Freely
β_{12}	6.644e-09 cm/W	Freely
β_{21}	6.644e-09 cm/W	Freely
β_{22}	2.349e-09 cm/W	Freely

Of particular note is the nonzero best-fit value for β_{11} in model 2. This appears to indicate that out-of-model nonlinear loss in the fundamental harmonic field can account for our measured data. Further study of the material properties of TFLN at these wavelengths and powers is needed to understand these dynamics and the processes contributing to them.

Supplementary Note 9. Poling of a shallower etched waveguide

The main advantage of our *pole-after-etch* method based on sidewall poling is that it results in complete (100%) poling of the entire waveguide cross-section, resulting in a high nonlinear conversion efficiency. For these results, shown in the main text, a 600 nm lithium niobate film was etched 520 nm. We also applied our approach to a 300 nm etched waveguide on a 600 nm lithium niobate film. After fabricating and poling the waveguide, the inverted domains are revealed using an SC1 etch (same process as discussed in the main text). From a scanning electron microscope image, Supplementary Fig. 12, we infer that full poling of the ridge can be achieved albeit at a higher voltage of 250 V. During earlier studies at these poling periods and with a 520 nm etch depth, these devices required about 150 V for full poling of the film. The poling period shown, approximately 3 µm, is tailored for second harmonic generation from infra-red to visible wavelengths.

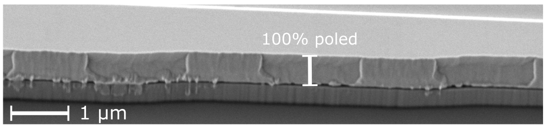

Supplementary Figure 12: Scanning electron microscope image of a sidewall poled TFLN waveguide with the inverted domains revealed by an SC-1 etch. To make this waveguide the 600 nm film is etched 300 nm instead of the 520 nm etch depth used for the main text results. At a poling voltage exceeding 250 V complete domain inversion of the film is observed.

Supplementary Note 10. Early exploration of narrow UV SPLN waveguides

Waveguides with 0.55 μm wide top width were fabricated (Supplementary Fig. 13a) to explore whether a higher intensity mode waveguide would result in higher conversion efficiency. The phase matching function for a 1.5 cm long device is measured (Supplementary Fig. 13) and follows a single peak, sinc^2 shape as expected from theory. We measure maximum on-chip UV power at 389 nm of 0.38 mW at 9.09 mW of pump power. This results in an absolute efficiency of 4.2%, about 6x lower when compared to the 1.75 μm wide waveguides reported in the main text. This is mainly attributed to the increased propagation losses measured for these narrower waveguides, 5.4 dB/cm vs 2.4 dB/cm. A diagonal etch where we reveal the inverted domains (see Methods main text), shows full film domain inversion, and consistent and straight domain walls (Supplementary Fig. 14).

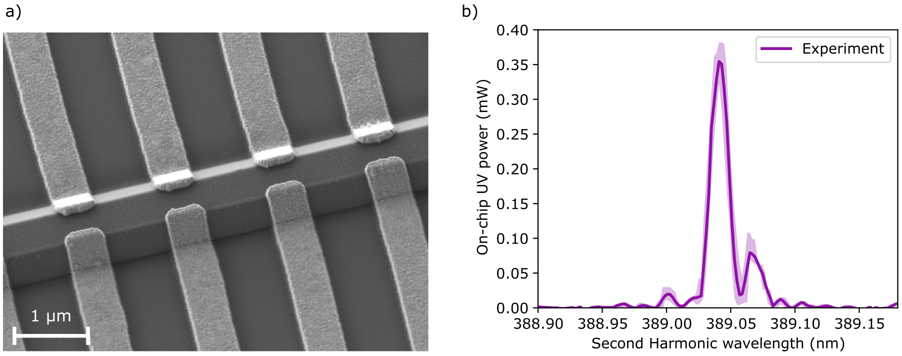

Supplementary Figure 13: a) Scanning electron microscope image of a narrower SPLN waveguide with a top width of only 0.55 μm. b) Phase matching function measured for a 0.55 μm wide and 1.5 cm long waveguide. The shaded area surrounding the solid lines denotes the experimental deviation in UV signal.

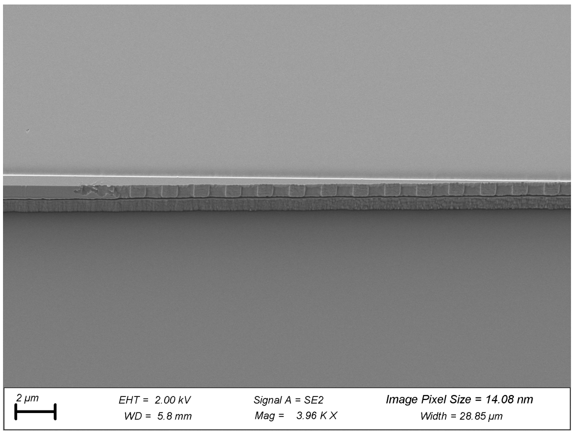

Supplementary Figure 14: SEM of a diagonal etch of a narrow (550 nm top width) poled waveguide, showing differentially etched poled domains (poling period 1.8 um, electrode duty cycle 35%, electrode tip-to-tip spacing 450 nm, poling voltage 105V). This waveguide underwent the same process described in Fig. 2d of our main work to reveal the poled domains. The duty cycle of the poling remains constant across the waveguide cross section, indicating straight and parallel domain walls despite the narrow waveguide width.

Supplementary Note 11. Simulations of poling field

The electric field applied to the LN waveguide through sidewall poling electrodes was modeled for several scenarios, including with and without an oxide interlayer, with short electrodes that did not touch the top of the waveguide, and with a narrow geometry (the experimental realization of this is documented in the previous section "Early exploration of narrow UV SPLN waveguides"). Visualizations of these simulations are provided here (Supplementary Fig. 15 to Supplementary Fig. 23), with further details in the captions.

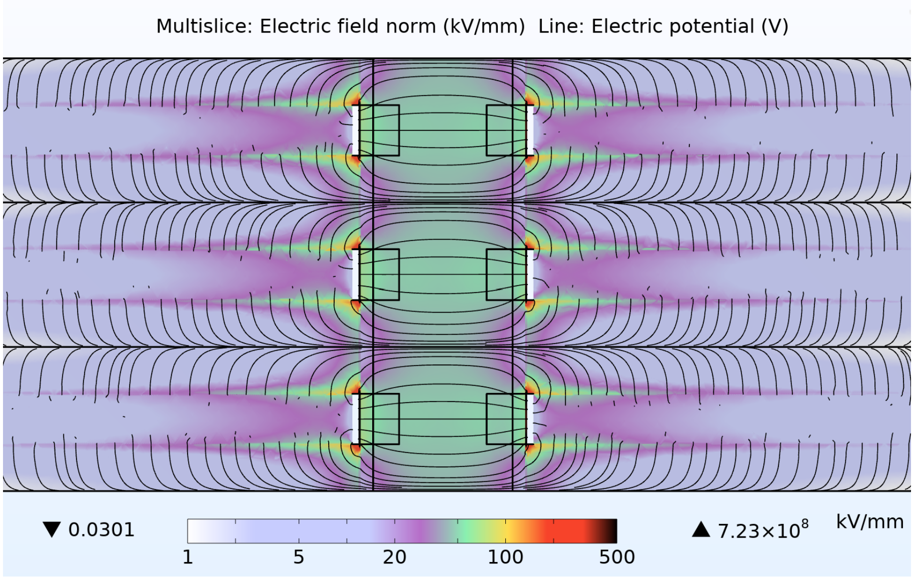

Supplementary Figure 15: Top-down view of poling field in nominal (with SiO2) geometry. Waveguide top width 1750 nm, poling voltage 127V, poling period 1.8 um, electrode duty cycle 35%.

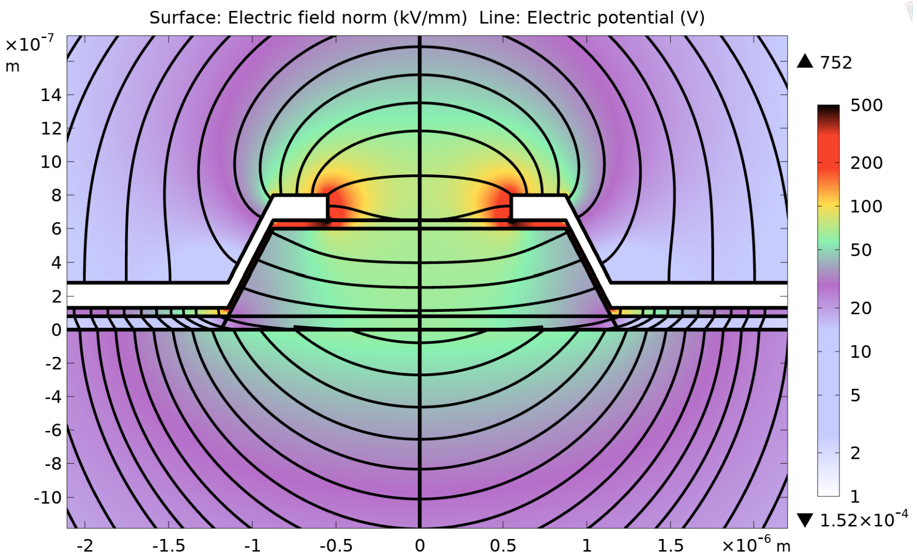

Supplementary Figure 16: Cross-sectional view of poling field in nominal (with SiO2) geometry. Waveguide top width 1750 nm, poling voltage 127V.

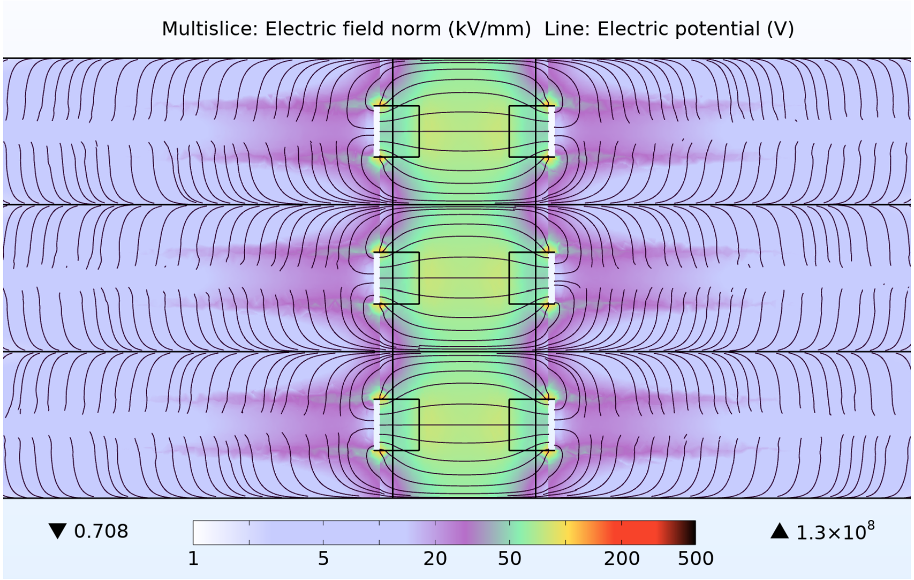

Supplementary Figure 17: Top-down view of poling field in no SiO2 layer geometry. Waveguide top width 1750 nm, poling voltage 127V, poling period 1.8 um, electrode duty cycle 35%.Note particularly the lower field value at the waveguide sidewall as compared with Supplementary Fig. 15.

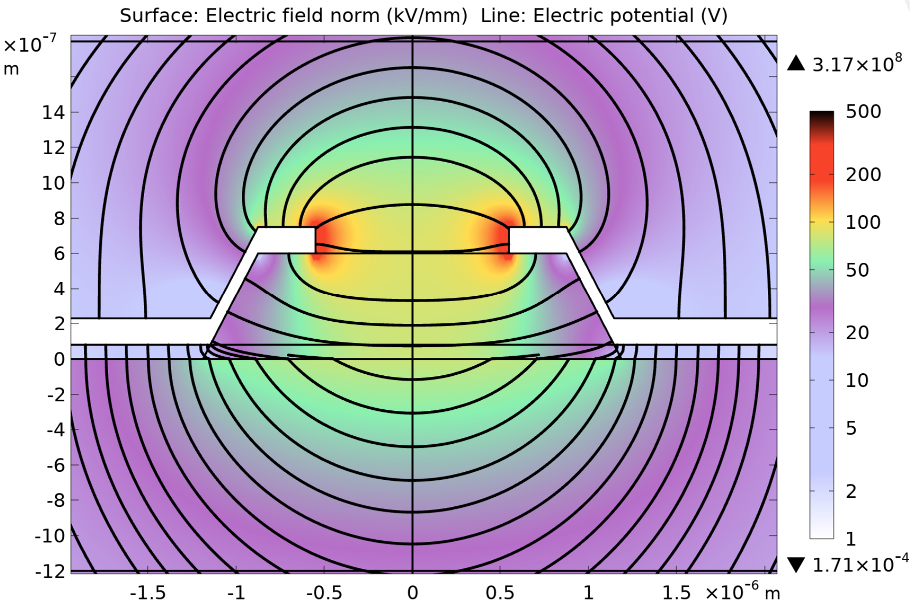

Supplementary Figure 18: Cross-sectional view of poling field in no SiO2 layer geometry. Waveguide top width 1750 nm, poling voltage 127V. Note particularly the lower field value at the waveguide sidewall as compared with Supplementary Fig. 16.

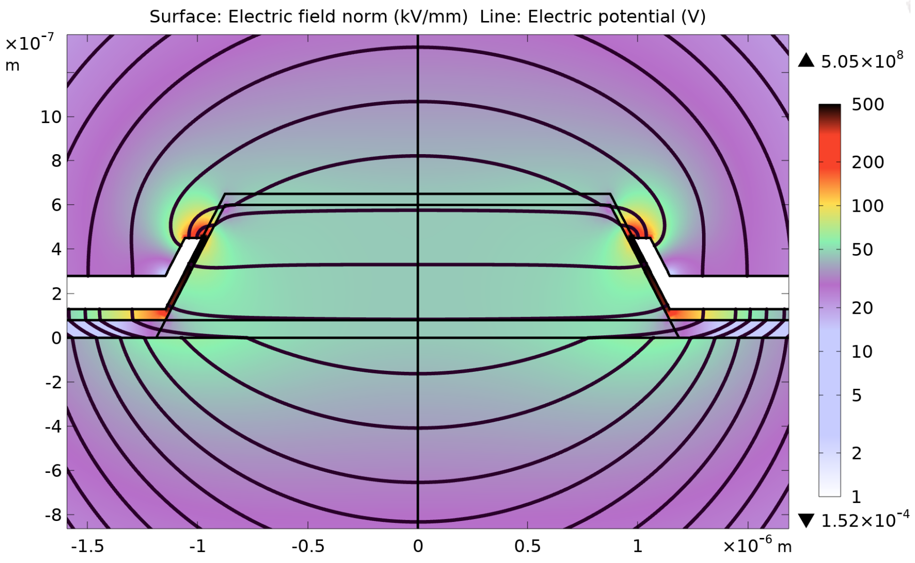

Supplementary Figure 19: Cross-sectional view of poling field in nominal geometry with short electrodes (which do not reach the top of the waveguide). Waveguide top width 1750 nm, poling voltage 127V. This impacts the uniformity of the poling field on the sidewalls of the waveguide, and may result in reduced domain formation at the top of the ridge.

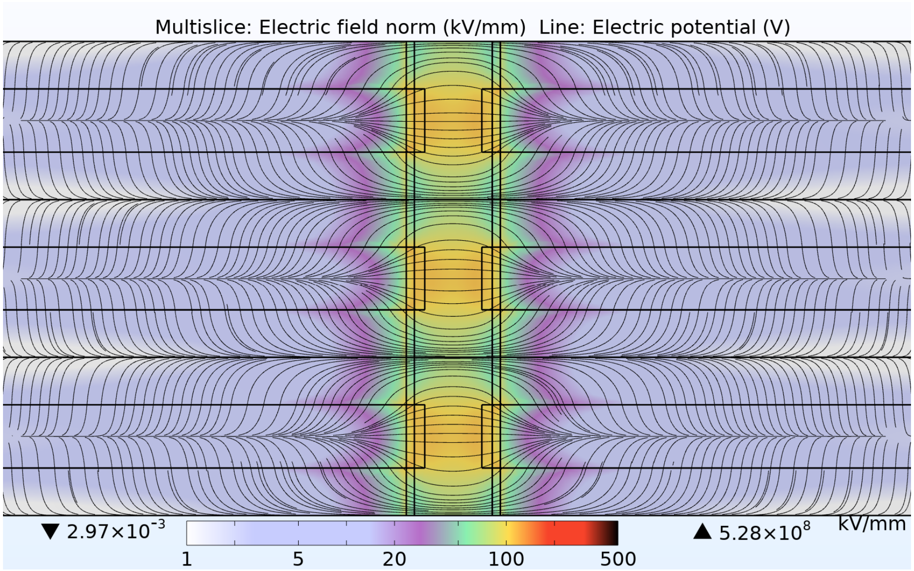

Supplementary Figure 20: Top-down poling field distribution halfway through the ridge of a shallow-etched (300 nm etch on 600 nm film) waveguide. Waveguide top width 1500 nm, poling voltage 250V, poling period 3.045 um, electrode duty cycle 40%.

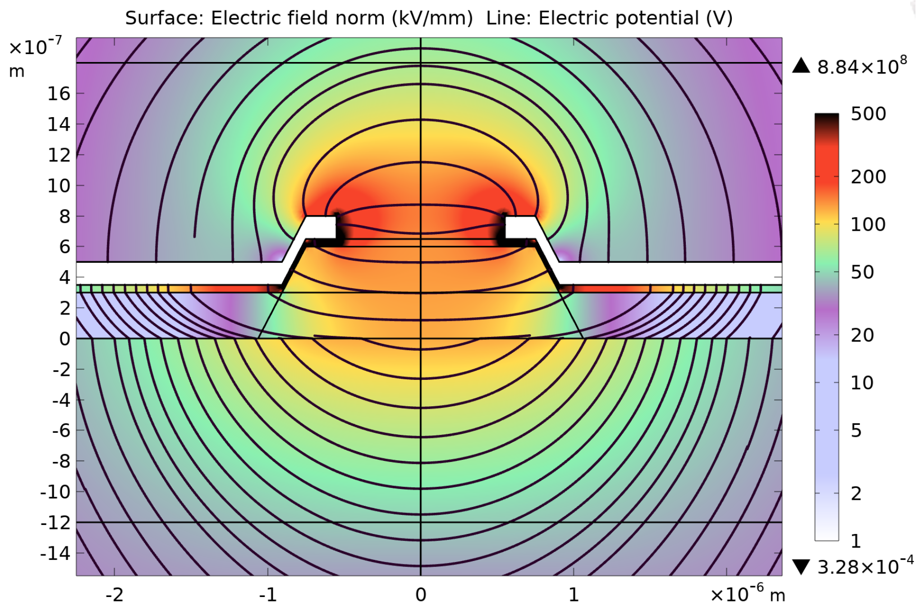

Supplementary Figure 21: Cross-sectional poling field distribution in a shallow-etched (300 nm etch on 600 nm film) waveguide. Waveguide top width 1500 nm, poling voltage 250V.

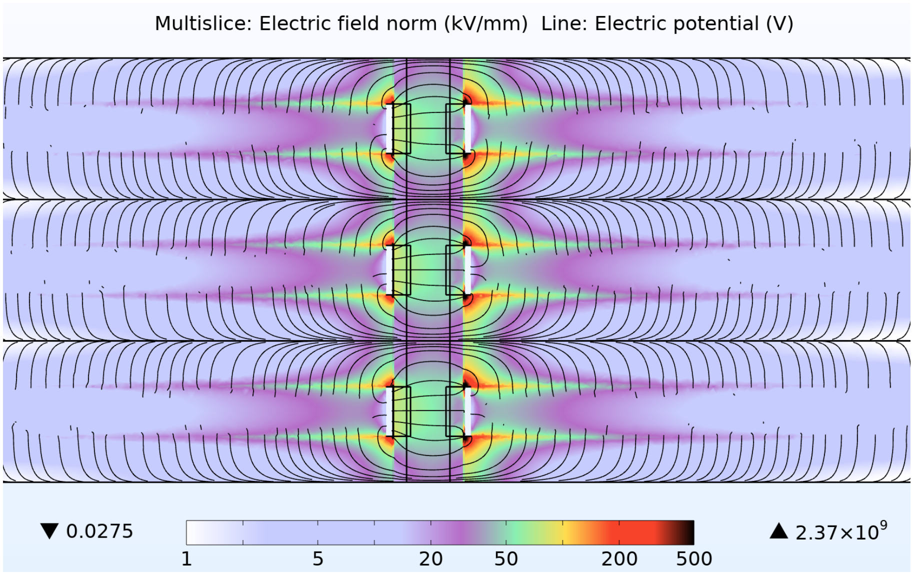

Supplementary Figure 22: Top-down view of poling field in a narrow (550 nm top width) waveguide. Poling period 1.8 um, electrode duty cycle 35%, electrode tip-to-tip spacing 450 nm, poling voltage 105V.

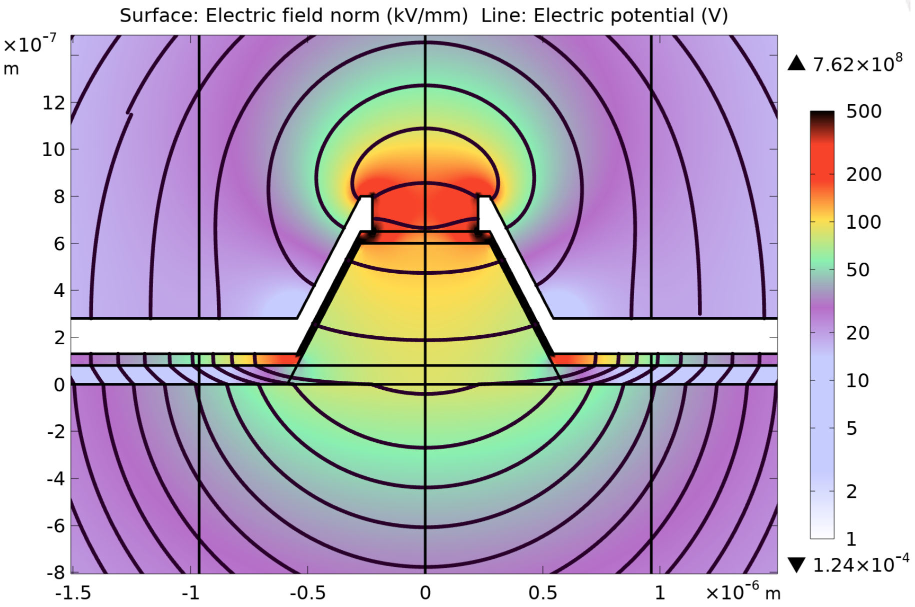

Supplementary Figure 23: Cross-sectional view of poling field in a narrow (550 nm top width) waveguide. Tip-to-tip electrode separation 450 nm, poling voltage 105V.

Supplementary References

[1] E. Hwang, N. Harper, R. Sekine, et al. "Tunable and efficient ultraviolet generation with periodically poled lithium niobate". *Optics Letters* 48.15 (2023). DOI: 10.1364/OL.491528.

[2] D. Melati, F. Morichetti, and A. Melloni. "A unified approach for radiative losses and backscattering in optical waveguides". *Journal of Optics* 16.5 (2014). DOI: 10.1088/2040-8978/16/5/055502.

[3] O. Beyer, D. Maxein, K. Buse, et al. "Femtosecond time-resolved absorption processes in lithium niobate crystals". *Optics Letters* 30.11 (2005). DOI: 10.1364/ol.30.001366.

[4] O. Beyer, D. Maxein, K. Buse, et al. "Investigation of nonlinear absorption processes with femtosecond light pulses in lithium niobate crystals". *Physical Review E* 71.5 (2005). DOI: 10.1103/physreve.71.056603.
